# Protein Recovery from Underutilised Marine Bioresources for Product Development with Nutraceutical and Pharmaceutical Bioactivities

**DOI:** 10.3390/md18080391

**Published:** 2020-07-27

**Authors:** Trung T. Nguyen, Kirsten Heimann, Wei Zhang

**Affiliations:** Centre for Marine Bioproducts Development, College of Medicine and Public Health, Flinders University, Health Science Building, Sturt Road, Bedford Park, Adelaide, SA 5042, Australia; trung.nguyen@flinders.edu.au

**Keywords:** seafood processing by-products, marine proteins, process and product development, nutraceutical and bioactive proteins, marine microalgae, biopeptides, enzymes

## Abstract

The global demand for dietary proteins and protein-derived products are projected to dramatically increase which cannot be met using traditional protein sources. Seafood processing by-products (SPBs) and microalgae are promising resources that can fill the demand gap for proteins and protein derivatives. Globally, 32 million tonnes of SPBs are estimated to be produced annually which represents an inexpensive resource for protein recovery while technical advantages in microalgal biomass production would yield secure protein supplies with minimal competition for arable land and freshwater resources. Moreover, these biomaterials are a rich source of proteins with high nutritional quality while protein hydrolysates and biopeptides derived from these marine proteins possess several useful bioactivities for commercial applications in multiple industries. Efficient utilisation of these marine biomaterials for protein recovery would not only supplement global demand and save natural bioresources but would also successfully address the financial and environmental burdens of biowaste, paving the way for greener production and a circular economy. This comprehensive review analyses the potential of using SPBs and microalgae for protein recovery and production critically assessing the feasibility of current and emerging technologies used for the process development. Nutritional quality, functionalities, and bioactivities of the extracted proteins and derived products together with their potential applications for commercial product development are also systematically summarised and discussed.

## 1. Introduction

The projected increase in protein demand has placed unprecedented pressures on protein production from traditional sources. Annual demand for dietary proteins of the present world population (7.3 billion) reached 202 million tonnes (MT) but this figure is predicted to increase to 1250 MT by 2050 [1,2]. Additionally, bioactive protein-derived products such as protein hydrolysates, and biopeptides have been shown to promote human health and have thus gained more importance in areas such as drug discovery, nutraceutical, and pharmaceutical developments [3]. The demand for these protein derivatives has steadily increased in line with expansion of the global market for nutraceutical and pharmaceutical products [4]. Commercial value of these therapeutic protein-based products in 2015 was USD174.7 billion and is predicted to reach USD266.6 billion in 2021 [5], leading to a two-fold increase in demand of protein-derived products. Production of such a large quantity of proteins to feed the whole world and to supply essential materials for nutraceutical and pharmaceutical developments using traditional methods requires intensive farming practices, which are associated with large demands of natural resources such as agricultural land, water, fertilisers, and feed. The problem is exacerbated by the recent steady increase in urbanisation, industrialisation, and ground water salt incursions in many regions of the world, which has led to a remarkable decrease in arable land. Furthermore, climate change as well as environmental problems have had additional negative impacts on global crop yields. It is therefore predictable that projected protein demands cannot be met through production from animals and plants alone [1,6]. Thus, alternative protein sources are needed to supplement existing sources in order to fill the demand gap for proteins.

**Figure 1:**
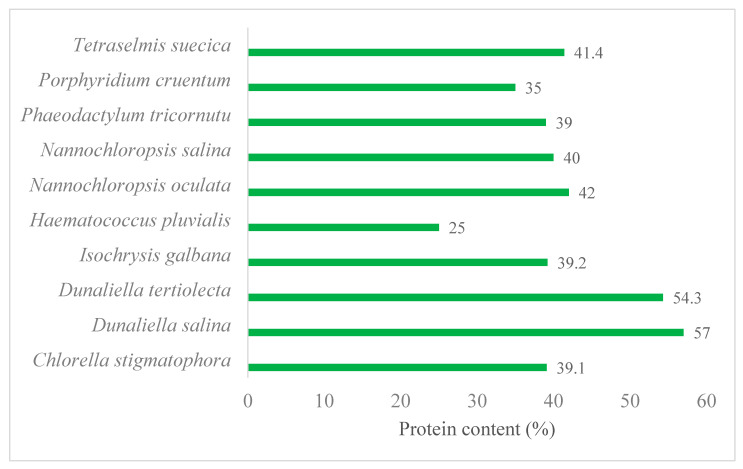
Protein contents of some typical marine microalgae.

Marine organisms represent a diverse and untapped bioresource for protein recovery and production. With an estimated 500,000–10 million marine species, the marine environment is characterised by an enormous biodiversity on approximately 72% of the Earth’s surface. It is hence not surprising that a trillion tonnes of marine fish, crustaceans, molluscs, and microalgae have been exploited for human consumption since ancient times. Abundant availability of fish processing by-products and technical advantages of microalgae production combined with their high protein contents make them ideal candidates for protein recovery. The global production of fish (freshwater and marine) is 171 MT, of which 108 MT are marine species [7]. However, by 2028 an increase to 178 MT is estimated, with 58% being predicted to be supplied by aquaculture [8]. Commercial seafood production only considers high value species and processing criteria fit for human food manufacturing, while the by-catch is discarded. At the processing stage, inedible and low-nutritional parts such as offal, heads, shells, skins, bones, trimmings, blood, and viscera are removed and discarded as biowaste. For this review, both the by-catch and the derived biowaste are defined as seafood processing by-products (SPBs), which can account for three-quarters of the total weight of production [9,10]. Annual production of marine SPBs is estimated at 81 MT, of which 32 MT are discarded as biowastes [11]. These SPBs, however, can contain up to 60% protein per unit dry weight of processed sea food [12]. They are therefore a hitherto underutilised bioresource for protein recovery. Similarly, protein contents of several marine microalgal species such as *Dunaliella salina*, *D. tertiolecta*, *Nannochloropsis occulata*, *Tetraselmis suecica*, *Tisochrysis lutea* (formerly *Isochrysis galbana* [13]), and *Chlorella stigmatophora* commonly range between 25–57% (Figure 1) [14,15]. Production of microalgal biomass can be flexibly conducted indoors or outdoors, depending on climatic conditions, on non-arable land with a minimal competition to conventional agricultural crops [16]. In addition, microalgal productivity is much higher than those of the conventional crops [17]. Readily abundant availability with no additional material cost for the SPBs combined with technical advantages in biomass production of the marine microalgae make these underutilised marine bioresources inexpensive and feasible for protein recovery to meet the increased demand for human consumption.

Marine proteins derived from the SPBs and marine microalgae are of high nutritional quality, good functionalities, and diverse therapeutic bioactivities have been demonstrated in a variety of commercial products. Regardless of biomaterial type used for protein production, the obtained products are typically protein concentrates (PCs) or protein hydrolysates (PHs). Enzymatic digestion of the PC and PH are being used as the sole or partial nitrogen source in specialised adult nutritional products and supplements [15,18,19,20]. Hydrolysis changes the chemical, physical, biological, nutritive, and immunological properties of proteins [21]. Following hydrolysis PHs are characterised by high nutritional quality attributed to high solubility and amino acid bioavailability, which positively affects gastrointestinal absorption rates, i.e., gastrointestinal absorption of di- and tripeptides are higher compared to other human-grade proteins and free amino acids [22,23,24]. Moreover, PHs contain a large amount of arginine, glutamic acid, glycine, and alanine which are responsible for their savoury taste making these protein products more palatable [25,26]. This, combined with their exceptional richness in essential amino acids, particularly methionine and lysine which are lacking in most cereals, corns, and legumes, make them superior to formulated protein products due to enhanced nutritional quality [27,28,29,30]. Such products also show promise for the treatment of malnutrition and anorexia [31,32,33]. Compared to casein-based diets, diets formulated with marine PHs have higher values of food efficiency ratios and net protein ratios [34]. Retained or even improved functionalities, such as solubility, water and oil binding, gelation, and emulsification of PCs and PHs are being used for production of sausages, fish balls, pate, patties, and pastes with high acceptability [35,36,37]. Excellent functionalities combined with intrinsic biocompatibility, bioactivity, and biodegradability have also been exploited for the formulation of nutraceuticals and pharmaceuticals [35,38,39,40]. Numerous marine-derived peptides with multiple nutraceutical and pharmaceutical bioactivities have been identified and reported. Some commonly encountered bioactivities of PCs and PHs are antihypertension, antioxidation, antibacterial, antidiabetic, immune-modulatory, and immune-stimulation [41]. For example, anti-hypertensive peptides from oysters and frames of Alaskan pollock (*Theragra chalcogramma*) [42], antioxidative peptides from Hoki frame [43], antidiabetic and anticancer peptides from Longtail tuna [44], and antiviral peptides from Winter flounder [45] have been extracted. Taurine and γ-aminobutyric acid (GABA) are amino acid derivatives with exceptional bioactivities. GABA is a natural neurotransmitter in the brain and functions in relaxation and improved sleep patterns [46], while the beneficial activities of taurine are far more divers (Figure 1). Bioactive amino acids such as taurine and GABA are found in the extracts and hydrolysed products of Alaskan pollock, southern blue whiting (*Micromesistius australis*), and the blood of some marine fish species [47,48,49].

Although several promising processes have been developed for the recovery and production of proteins from SPBs and marine microalgae, more efforts on process and product development are still required to make their commercial production economically feasible [1,50]. Mechanical processes used to recover protein-rich meats or muscles from frames, heads, or shells of the SPBs can be deployed for disrupting microalgal cell walls to facilitate protein extraction. Isoelectric solubilization/precipitation (ISP) is a common method suggested for recovery of fish-SPBs and microalgal protein concentrates [38,51]. Solvent and enzymatic extraction processes are preferred for production of protein products with improved nutritional quality, functionalities, and bioactivities [52]. Although the efficiency of recovery and production of marine proteins from the underutilised bioresources has been demonstrated for some processes, industrial applications for sustainable production is still not feasible due to multiple steps, low yield, and high production costs [53,54]. Hence novel and simpler processing strategies are required to reduce operational costs. Such processing strategies should be intensified to improve production yields and retain and/or improve nutritional quality, functionalities, and bioactivities to generate high-value commercial products. This review comprehensively analyses the potential for commercial production of marine proteins from SFBs and microalgae by discussing material availability and conventional technologies used in process development. Commercially interesting bioactivities of the protein products with their nutritional quality and functionalities for nutraceutical and pharmaceutical products are discussed. Furthermore, the feasibility of current and emerging technologies used for process development are critically assessed.

## 2. SPBs and Marine Microalgae as Advantageous Bioresources for Green and Sustainable Production of Proteins and Protein-Based Products

Despite being presently underutilised for production of biofertilizer and animal feeds with low economic profitability or even discarded as biowastes with a considerable disposal cost and environmental problem, several SPBs and marine microalgae are rich in proteins. This offers potential for the development of protein-derived products for human consumption and several other advanced applications through recovery from the underutilised resources.

### 2.1. Inexpensive and Untapped SPBs for Recovery of Various Proteins and Protein Derivatives

#### 2.1.1. Heads, Shells, and Frames for Recovery of Muscle Proteins, Carotenoproteins, and Their Hydrolysed Products

Heads, shells, and frames are inedible parts which are often separated and discarded as waste during the processing of several fish products such as fish fillets, fish meats, pastes (surimi), salting, smoking, and canning. The inedible parts constitute the largest proportion of SPBs, but ratios vary widely depending on species and the processing process (Table 1). For example, the range of heads, shells, and tails in commercial processing of crustacean ranges from 45% to 60% [55,56], but constituted 80% in high hydrostatic processing of American lobsters [57]. Waste amounts are lower for other species; for instance, mollusc 43% and finfish 25–35% [58]. Annual production of these SPBs from Scottish salmon processing alone was 76,052 tonnes in 2015 [59] while quantity generated by the lobster industry of American, Canada, and Australia produced 50,000 tonnes [60]. In contrast, SPBs of the crustacean processing industry including crab, shrimp, and lobster account for 6–8 million tonnes [56]. Presently, about 40% of these SFBs is used for the production of low-value biofertilizer and animal feed products [61], with most SPBs being landfilled or disposed of as biowastes with high disposal costs [62]. Considering the financial and environmental burden, sustainable recovery of proteins from residual meats or muscles as rich-protein sources for sustainable recovery should be possible.

Indeed, shells of crustacean species usually contain significant amounts of proteins; 25% in Australian Southern Rock lobster shells [18], and 20–40% in shrimp and crab shells [56], but protein content can also be as low as 5% in mollusc shells [91]. In contrast, heads and frames generated in the seafood processing industry often contain a considerable amount of residual meat or muscle, i.e., up to 20% of the lobster weight including body, breast, and leg meats [92]. As a result, 43.5% protein is found in lobster heads such as Australian Southern Rock lobster while this content of heads and frames of finfish such as catfish are 47.3% and 32.5% [93], and 12.9% and 16.4% for gilthead [65]. Such quantities demonstrate that heads, shells, and frames are resourceful and inexpensive bioresources for protein production and recovery, but economic feasibility is likely restricted to species with high protein contents in the discarded materials, because different feedstock are likely to demand utilization of differing extraction, denaturation and temperature regimes, making centralization of processing difficult to achieve. In regions, however, where sufficient amounts of different SFBs are available at different times of the year, a centralised facility could offer significant economic advantages.

#### 2.1.2. Skins, Scales, and Bones for Recovery of Collagens, Gelatines, and Their Hydrolysed Products

Skin, scales, and bones are usually separated as inedible parts in several fish processing processes. These parts could make up as high as 30% of fish processing by-product [65,68]. With 54.1 million tonnes of finfish being annually caught [93], an estimation of 16.2 million tonnes of fish skins, scales, and bones are globally produced each year. Experiencing the same status as other SPBs, these fish skins, scales, and bones are presently underutilised, however, they are protein-rich sources (Table 1). For instance, fish scales contain 30–50% protein [94], but very high amounts are found in the dried scales of mullet species, e.g., 78.1% in *Liza macrolepis*, 70.4% in *Liza melinoptera*, 76.5% in *Mugil cephalus*, and 62.3% in *Valamugil speigleri* [71]. Protein in fish skins is lower, 20.1% in blue shark (*Prionace glauca*), 22.1% small-spotted catshark (*Scyliorhinus canicula*), 27% in yellowfin tuna (*Thunnus albacares*), and in 16.3% swordfish (*Xiphias gladius*) [68]. For the same fish species, proteins in fish skin is higher than in bone, e.g., gilthead sea bream skins contain 24.8% protein but only 16.4% are present in its bones [65]. Importantly, however, these by-products are the main sources of structural and fibrous protein known as collagen which is the most abundant protein in mammals and the major constituent in connective tissues, skins and bones of animals. Collagen contents of the SPBs vary widely depending on the species, age, and season and can represent up 70% of dry weight for some specific species [68], e.g., 71% in leather jacket mince (skins, bones, and muscles) [95] or 78.6% in southern catfish (*Silurus meridionalis Chen*) skin [96]. Therefore, skins of different marine fish species such as bigeye snapper (*Priacanthus macracanthus*, *Priacanthus tayenus*, *Priacanthus hamrur*) [97,98,99] and cuttlefish (*Sepia pharaonis*) [100] have been utilised for extraction of collagen and its derivative gelatine, with the yield ranging from 10.9 to 33.2% on average. Extraction yields can, however, be much higher for some other species such as ocellate pufferfish 44.7% [101], squid 53% [102], and brown backed toadfish 53.4% [103]. Fish bones also contain a significant amount of collagen, i.e., in some fish species 42.3% (skipjack tuna), 40.7% (Japanese sea bass), 53.6% (ayu), 40.1% (yellow sea bream), and 43.5% (horse mackerel) [104] but only 1.6% in bigeye snapper (*Priacanthus tayenus*) [105]. Fin collagen (on the basis of lyophilized dry weight) of Japanese seabass was 5.2% acid-soluble collagen and 36.4% acid-insoluble collagen [104] while for tuna fin this was only 2% [106]. Collagen was also extracted from scales of several marine fish species, for example, lizard fish (*Saurida* spp.), horse mackerel (*Trachurus japonicus*), grey mullet (*Mugil cephalis*), flying fish (*Cypselurus melanurus*), and yellowback seabream (*Dentex tumifrons*); however, their yields (on dry basis) were low, only 0.43–1.5% [107]. The above demonstrates that fish skins, scales, bones and fins would represent great sources for protein recovery and are also promising alternatives for collagens and gelatines.

#### 2.1.3. Viscera for Production of Intestinal Enzymes and Biopeptides

Viscera is completely removed during fish processing since this part contains several biologically active components that could negatively impact on the on quality of postharvest fish products. As shown in Table 1, the whole visceral parts including livers, stomachs, spleens, and roes account for 12–18% of total finfish volume [108] whereas amounts for only fish viscera are 2–8% [109]. With 80 million tonnes of fish globally produced per year, annual production of viscera is estimated to be 11.5–29.7 million tonnes [110]. Protein contents of viscera generated from different fish species range between 8.5–21% [65,109], but is extraordinarily high in tuna viscera (65%) [108], being comparable with the edible parts or flesh. Therefore, visceral by-product has been identified as a valuable source for recovery of native proteins, protein hydrolysates, and biopeptides [108,111]. Moreover, viscera-derived SPBs represent rich sources of diverse proteases and several other enzymes (chitinase, alkaline phosphatase, and hyaluronidase) which are abundantly available in the intestines followed by pyloric ceca, pancreatic tissues, hepatopancreas, shell, and other waste components [112]. A variety of enzymes recovered from different SPBs of various fish and shellfish species have been reported [113]. Thus, the potential of fish and shellfish viscera for recovery of proteases is large [114]. Some examples of commonly extracted gastric, intestinal and hepatopancreatic proteases are pepsin trypsin, chymotrypsin, collagenase, and elastase [115], followed by non-proteolytic enzymes, such as transglutaminase, lipases, and chitinolytic enzymes obtained from various fish species, such as the Nile tilapia (*Oreochromis niloticus*) [116] or skipjack tuna (*Katsuwonus pelamis*) [117]. Therefore, visceral by-products have been evaluated as a favourable source of gastric, intestinal, and hepatopancreatic enzymes [118,119,120]. Trypsin has been successfully extracted from viscera of different commercial fish species such as *Reochromis niloticus* [121], *Lutjanus vitta* [122], and *Katsuwonus pelamis* [123] with a promising yield of 22.1% [124], making the process suitable for commercial scales with yields of 1–3 g of purified trypsin per kilo of wet waste. The extraction of nutraceutical proteins and isolation of commercial enzymes from the visceral by-product may introduce a valid strategy for efficiently transforming a costly and polluting waste source into a profitable product range.

#### 2.1.4. Fish Blood for Production of Protein Hydrolysates, Biopeptides, Bioactive Amino Acids (Taurine, GABA), Protease Inhibitors, and Cell-Culture Media

Removal of blood is the first important step in several seafood processing pathways since quick coagulation and oxidation of fish blood negatively affect product quality. After bleeding, fish blood is completely washed away generating the blood-water waste stream which poses a substantial environmental problem and economic cost for seafood processors [73]. Blood volumes in live fish range from 2 to 7% of a fish’s body weight (Table 1), but the range for salmon and trout is higher (3.5–4%). Around 2% fish blood was recovered from the Norwian salmon processing industry, equating to approximately 26,000 tonnes in 2015 [73]. Therefore, opportunities for utilisation of fish blood are large because this waste stream contains different proteins with contents between 0.9–5.7% [74], providing potential source for production of protein hydrolysates, biopeptides, and active amino acids for high-value add co-products [73]. Fish blood plasma contains a variety of protease inhibitors [125,126,127,128], including α2-macroglobulin, a protein that inhibits several classes of proteases through a bait and trap mechanism [129]. In addition, fish plasma protein is a promising alternative to foetal bovine serum in cell culture media for cell tissue cultures [130].

### 2.2. Marine Microalgae as an Advantageous Biomass for Green and Sustainable Production of Proteins and Enzymes

The use of marine microalgae for production of proteins and enzymes has recently attracted increased attention since microalgal biomass has several practical advantages. Marine microalgae are considered to represent the largest primary biomass in our oceans [131]. Microalgal biomass can be environmentally sustainably produced with minimal competition with traditional food crops for areal space and resources. For example, production of 1 kg microalgal biomass requires less than 0.25 m^2^ of land and 0.5 m^3^ of water while production of the equivalent weight of beef are up to 7.9 m^2^ and 15.5 m^3^, respectively [132,133]. As primary producers, microalgae fix CO_2_ (autotrophy) and other organic carbon sources through mixotrophy and/or heterotrophy to synthesise proteins, bioactive metabolites, and lipids. Particularly, growing microalgae for biomass production can be flexibly conducted in bioreactors under optimal conditions indoors or outdoors where land and oceans are underutilised to minimise impact on scarce or dwindling agricultural production surfaces [134]. Furthermore, microalgae biomass can also be sustainably produced by integrating wastewater treatment and atmospheric carbon dioxide sequestration for autotrophic clean production [135]. Other than environmental-friendly and sustainable production, economic feasibility is substantial because of high productivities, i.e., annual production yields of some species can reach up to 250 tonnes/ha [136], which is several times higher than those of any other agricultural commodity [137]. Particularly, achieved protein production yields are with 4–15 tonnes/ha significantly higher than for crops (e.g., 1.1, 1–2, and 0.6–1.2 tonnes/ha for wheat, pulse legumes and soybean, respectively) [138]. Microalgal production is seriously being evaluated as an unconventional source of protein. As pointed out previously (Figure 1), many microalgal species are rich in protein with contents ranging from 25–57% depending on species and cultivation phase (i.e., protein contents are typically highest in exponential growth phase and reduce in stationary phase), much higher compared with conventional sources [14]. The commercial production of microalgal biomass from marine species such as *Dunaliella salina* has been developed in several countries (USA, Israel, Australia, China, and Thailand) since 1980 [139]. A large quantity of different enzyme classes, such as hydrolases, oxidoreductases, and lyases involved in light harvesting, carboxylation, glycolate-metabolism and protection from reactive oxygen species [140], such as cellulases, galactosidases, proteases, lipases, phytases, laccases, amylases, and antioxidant enzymes, among many others have been identified in microalgae [141]. The above mentioned significant advantages of microalgal production combined with demonstrated nutraceutical and pharmaceutical bioactive compounds and enzymes renders this biomass as a promising resource for commercial production of proteins and enzymes [142,143,144,145].

## 3. Nutritional Quality and Biological Activities of Marine-Derived Proteins and Their Derivatives

### 3.1. Nutritional Quality of Marine Proteins

Microalgae and fish proteins are considered excellent sources of functional and bioactive nutrients for human nutrition requirements, but whole cell microalgal diets contain cell walls, membranes, and polysaccharides limiting digestibility [146]. In contrast, extracted microalgal proteins are readily available for digestive enzymes improving digestibility to 82% [147]. Similarly, marine fish proteins with low amounts or an absence of stroma (collagen, elastin, and gelatine), collagenous fibres and tendon proteins are tender and easily digestible resulting in high absorbability and bioavailability. The in vivo digestibility of proteins derived from raw finfish and shellfish is in the range of 85% to 98% [148]. Indeed, proteins enzymatically extracted from southern rock lobster shells had a digestibility of 96.9% while ultrasound-intensified ISP extraction of lobster head proteins was 78.4% [19,55]. Seafood proteins are high quality, contain sufficient amounts of essential amino acids (EAAs) required to assure healthy growth and development of humans in addition to supporting human health in general. Marine microalgae proteins have a well-balanced amino acid profile, in which EAA contents of some species such as *Dunaliella salina* and *Nannochloropsis salina* were up to 48–51%, higher than those of freshwater species, i.e., *Chlorella* sp. and *Spirulina* sp. (44.4% and 43.8%) [15]. Marine fish proteins also have a competitive percentage of EAAs, 45.6% in proteins of finfish species, e.g., yellowfin tuna [149] and 38.9–41% in proteins of crustaceans, e.g., lobster and shrimp [19,150]. The EAA profiles of marine proteins are comparable to those of milk and eggs with only methionine being a limiting amino acid (e.g., 0.66 for microalgal proteins of *Dunaliella bardawil* and 0.85, 0.76 for fish or lobster proteins, respectively) [146]. However, microalgae and SPB-derived proteins are far superior to those of soybean and proteins derived from other plant sources [136,151,152]. Their exceptional richness in arginine, accounting for 13.4 and 10.8% in proteins of *N. salina* and *D. salina*, respectively [153] highlight their nutraceutical and pharmaceutical potentials, as this amino acid plays a vital role in protein synthesis, detoxification, and energy conversion [154] and has been recommended for the treatment of cardiovascular disease [155]. Proteins of several marine fish are also rich in arginine. Lysine/arginine ratios have been used for evaluation of the nutritional value of proteins, since consuming proteins with a high ratio causes some lipidemic and atherogenic effects [156]. The very low lysine/arginine ratio of marine proteins, e.g., 0.16–0.42 for microalgal proteins (*N. salina* and *D. salina*) [15] and 0.7–1.0 for crustacean proteins (lobster shells and heads) [18,19] compared to 13.8 in meat proteins [157] highlights potential health benefits when including marine proteins in human diets. In addition, fish protein characterised by enrichment with savoury aromatic amino acids together with nonprotein nitrogen compounds (i.e., small peptides, trimethylamine oxide, trimethylamine, creatine, creatinine, and nucleotides) enhances the palatability of a wide variety of foods thereby increasing product potential [158,159,160,161,162]. Therefore, fish proteins are commonly used to improve the palatability of diets or fortify the overall protein content of cereal-based diets, which generally lack some essential amino acids.

### 3.2. Bioactivities of Marine-Derived Proteins

Fish proteins contain various bioactive peptide sequences which become active after being hydrolysed. These biopeptides are released from parent proteins during normal gastrointestinal digestion or during food processing with the use of heat, chemicals, proteolytic enzymes or microorganisms (fermentation) [163]. Due to having beneficial modulatory functions for some metabolic pathways, these biopeptides may play a vital role in disease prevention and health promotion. Their biological activities are largely determined by their structural properties such as molecular weight and the physico-chemical characteristics of the amino acids within the sequence [164]. For the production of bio-peptides via hydrolysis, variable factors such as pH, time, temperature, the enzymes used, and the enzyme-to-substrate ratio strongly affect the bioactivities of the generated protein hydrolysates and biopeptides [165,166]. To produce bioactive peptides with high bioactivities, these factors should be carefully controlled. Amino acid sequences determine protein structure and function. Therefore, different proteins have diverse molecular properties. i.e., fibrillar collagen, sarcoplasmic, stroma, gelatine, and plasma from different sources (microalgae, finfish, crustaceans, molluscs, and coelenteratae) would generate numerous types of peptides with a variety of bioactivities. The biological activities of the released peptides differed for each source due to the initial protein source, the enzyme employed, and the processing conditions used [167].

#### 3.2.1. Antihypertensive

Antihypertensive effects of protein hydrolysates and their peptides can be evaluated by measuring Angiotensin-I-converting enzyme (ACE) inhibitory activity in vitro or activity in spontaneously hypertensive rats (SHR) in vivo [168]. Numerous marine-derived protein hydrolysates and peptides have been evaluated using the ACE inhibitory activity assay and in spontaneously hypertensive rats [168,169,170,171]. ACE inhibitory peptides are generally short sequences with molecular weights ranging 300–3000 Da, containing 2–13 amino acid residues including Gly, Tyr, Leu, Arg, Asn, Thr, Asp, Trp, Val, His, and Phe [167,172]. The optimum amino acid residues for potent ACE inhibition analysed in silico are peptides starting with Tyr and Cys in the first position at the C-terminus; Trp, Met, and His in the second position; Leu, Ile, Val, and Met in the third position, and Trp in the fourth position [173]. Peptides with hydrophobic and aromatic residues at N- and C-termini, usually exert strong anti-hypertensive activity [174]. In vitro, the inhibitory potency of peptides is expressed as the IC_50_ concentration [175], the peptide concentration which inhibits 50% of the ACE activity. Most reported anti-hypertensive peptides of marine proteins have ACE IC_50_ values ranging 0.3–1500 μM [176]. The ACE IC_50_ values of marine microalgal peptides (e.g., *Nannochloropsis oculata*, *Chlorella ovalis*, *Chlorella ellipsoidea*, *Nannochloropsis oculata*, and *Tisochrysis lutea*) range from 0.6 to 236.9 μM [170,177,178,179,180] and those of proteins derived from SPBs are 3.07–35.7 μM [167], comparable to milk (2–315 μM) [181] and meats (0.21–945.5 μM) [182,183], but higher than for egg (4.7 μM) [184] and soybeans (0.082–8.5 μM) [185]. Among fish species, the ACE IC_50_ values range from 0.4 to 105 μM for finfish (Bonito, Alaska pollack, flounder) [186], 0.9–24.1 μM for crustaceans (shrimp, krill) [187], 1.2–51 μM for molluscs (oyster, clam, squid) [188,189], and 2.4–23.4 μM for coelenterates and echinoderms (jelly fish 8.4–23.4 μM [190], and 2.9–9.1 μM sea cucumber [191]) (Table 2). These bioactive peptides were found to be resistant against several gastrointestinal digestive proteases including pepsin, trypsin, α-chymotrypsin, and pancreatin, suggesting that their inertness is essential to exert their anti-hypertensive effects at the active site [192,193,194]. Many anti-hypertensive peptides exhibited activity in vitro also exerted strong activity in vivo. For example, dosages of 10 mg tuna frame/kg of body weight and 3 μmol sea cucumber peptides/kg of body weight reduced systolic blood pressure (SBP) to 21–25 mmHg [168] and 17 mmHg on average, respectively [191].

Anti-hypertensive activity of marine proteins and hydrolysate is also attributed to the richness of biologically active amino acids. Taurine, which acts as an osmostress protectant in many marine organisms, is usually present in high amounts in marine species, especially in invertebrates such as molluscs and crustaceans [207]. Taurine levels of 655, 70, and 240 mg/100 g wet weight were found in raw mussel, fresh oysters and clams, respectively, while 151 and 31 mg/100 g wet weight were reported for raw white fish and frozen cod, respectively [208]. The extracts and hydrolysates of Atlantic salmon, Coho salmon, Alaska pollack, and southern blue whiting were reported to contain 27, 19.5, 15.2, and 149 mg of taurine/100 g dry weight, respectively [48]. Taurine accounted for 0.8–1.8% of crude proteins derived from several marine microalgal species (e.g., *Heterocapsa rotundataa*, *Ansanella graniferaa*, *Alexandrium andersoniia*, and *Gymnodinium smaydaea*) [153]. Taurine is an active amino acid participating in several essential biological processes such as calcium modulation, bile acid conjugation, antioxidation, membrane stabilization, and immunity [49,208]. Taurine has been reported to perform anti-hypertensive activity in both rat and human studies [208,209,210]. Another active amino acid-like molecule is GABA which can be synthesised from glutamate by two glutamic acid decarboxylase enzymes [211]. GABA was detected in high amount in the intracellular amino acid pool in erythrocytes of flounder, plaice and dab [47]. GABA accounted for 3.1–5.8% in crude protein of several marine microalgal species [153]. GABA has been reported to participate in the regulation of nearly all main developmental steps from cell proliferation to circuit refinement [212]. It reduced blood pressure in the SHRs [213] and mild hypertensive subjects [47].

#### 3.2.2. Antioxidant

Proteins, hydrolysates, and peptides derived from marine microalgae and fish possess antioxidative activity via a variety of mechanisms. The two best known mechanisms are prevention of oxidative damage by interrupting the radical chain reaction of lipid oxidation and scavenging. Antioxidant scavenge free radicals (FRs) and reactive oxygen species (ROS) [214,215,216]. Fish proteins significantly lowered lipid peroxidation in spontaneously hypertensive rats (SHRs) compared to casein. Hence, fish proteins were assumed to play an important role in the antioxidative defence system of some organs such as heart and liver, but not in plasma [217]. However, a significant increase in antioxidant status compared to controls (casein diets) was demonstrated in blood and plasma of SHRs models with diabetes [218]. Additionally, proteins of fish species such as cod and scallop had beneficial metabolic effects, reducing atherosclerotic plaque burden, serum glucose, leptin, and low density lipoprotein cholesterol levels [219]. Antioxidant effects of fish proteins were also observed in human nutritional intervention trials. Consuming fish proteins significantly increased plasma antioxidant status and considerably reduced in the amount of the oxidation products (circulating malondialdehyde) which was accompanied by weight loss of 2.6–9%. Compared to lean-meat and the lean-meat-omega-3 nutritional groups, the antioxidative capacity was significantly increased in the fish-protein group, suggesting they may be a useful strategy to lose weight and reduce oxidative stress [220]. Proteins enriched in histidine, glutamic acid, aspartic acid, along with phosphorylated serine and threonine may increase inhibition of lipid oxidation significantly due to the ability of these amino acids to chelate pro-oxidative transition metals [221].

Hydrolysates and peptides derived from microalgal and fish proteins exhibited antioxidant activity in many studies. Protein hydrolysates of marine microalgae, e.g., *Navicula incerta*, demonstrated strong free radical scavenging activity on 2,2-diphenyl-1-picrylhydrazyl (DPPH), hydroxyl, and superoxide radicals with the IC_50_ values of 0.49–0.94 μM [222]. Based on DPPH scavenging and ferrous chelation, an IC_50_ range of 1.54–8.54 μM were also reported to fish protein hydrolysates produced from by-products of different fish species such as sardine, horse mackerel, bogue, and small-spotted catshark [223]. Numerous antioxidative peptides derived from various marine microalgal and fish species are summarised in Table 3. Antioxidant peptides were produced from proteins with hydrolysis degrees of 13–75%. Their molecular weights ranged between 200–2000 Da, containing 5–16 polar and hydrophobic amino acids commonly comprised of Asp, Glu, Gly, Ala, Leu, His, Pro, Tyr, Phe, and Lys. However, the specific contribution of each individual amino acid to the antioxidant activity of a peptide depends largely on the nature of the ROS/FRs and the reaction medium [164]. Antioxidant activity of any protein hydrolysate or peptide primarily depends on the process parameters which determines its structural properties [224]. A peptide sequence with a high amount of hydrophobic residues at the N terminal position accompanied with aromatic, amphiphilic or polar amino acid residues at C terminal positively enhance antioxidant properties [46]. The presence of hydrophobic amino acids in peptide sequences is believed to vitally contribute to their antioxidant potency [225]. Gelatine peptides are expected to exert higher antioxidative activity compared to many others due to dominance of hydrophobic amino acids favouring emulsification and inhibitory reactions [224]. In fact, inhibition of lipid peroxidation by peptides produced from skin gelatine of jumbo squid using a linolenic acid model system was much higher compared to tocopherol and comparable to a commercial synthesized antioxidant, BHT [214]. Therefore, bio-peptides produced from proteins of marine species such as collagens and gelatines of fish skins, scales, bones, and fins could potentially replace synthetic antioxidants.

#### 3.2.3. Antidiabetic

Proteins from different sources play vital roles in glucose metabolism. These active roles of microalgal and fish proteins in the regulation of serum glucose levels have been highlighted in several intervention studies [237,238,239,240]. Intake of fish protein diets for 6–8 weeks significantly reduced serum glucose levels in rats compared to casein controls [239]. The significance of fish proteins in antidiabetic effects compared with those of proteins from other sources (beef, pork, veal, eggs, and milk) was also highlighted in a human intervention study, as cod protein-supplemented diets led to improved insulin sensitivity in overweight or obese males and females in a 4-week period [237]. Such activities have also been reported for protein hydrolysates and peptides derived from microalgal and fish. Feeding alloxan-induced diabetic rats with microalgal and fish protein hydrolysates significantly decreased malondialdehyde (MDA) levels and delayed the occurrence of diabetic complications [241,242,243]. The actual underlying mechanism(s) of these antidiabetic effects are not yet fully understood, but dietary amino acids and short peptides derived from these proteins were assumed to be involved in the regulation of glucose metabolism in a number of ways. These include direct stimulation of pancreatic cells to secrete insulin, inhibition of metabolic enzymes such as dipeptidyl peptidase IV (DPP-IV) and α-glucosidase which are involved in the regulation of serum glucose, stimulation of secretion of incretins (i.e., glucose-dependent insulinotropic polypeptide (GIP) and glucagon-like peptide-1 (GLP-1)) [244]. Many bioactive peptides with antidiabetic potency have been isolated from microalgal and fish protein hydrolysates (Table 4) [211,245,246]. Bioactive peptides with potent inhibition of DPP-IV have specific peptide motifs that consist an N-terminal Trp and/or a Pro at position 2 [245]. These bio-peptides inhibit DPP-IV at IC_50_ concentrations of 0.02–8139.1 μM. Peptides derived from fish collagen were found to exhibit high DPP-IV inhibitory activity with low values of the IC_50_ (0.02–0.07 μM) [202], while peptides derived from allophycocyanin and phycoerythrin, abundant in several marine cyanobacteria and red algal species [247], assumed to have a high potency index [248], had the IC_50_ concentration of 10.9–12.9 μM. Different action modes and various engagement routes for regulating glucose metabolisms by antidiabetic peptides have been identified [244,245]. For example, by down-regulating oxidative stress and inflammation related to type-2 diabetic mellitus (T2DM) and protecting pancreatic β-cells from apoptosis, salmon skin peptides exerted strong antidiabetic effects in T2DM rats [249]. Bioactive peptides, which exhibited DPP-IV inhibitory activity in vitro and animals, also had strong antidiabetic effects in diabetic patients [238].

#### 3.2.4. Anticancer

Free amino acids have been reported to have diverse effects in various cancer cells [253,254]. Glutamic acid induced apoptosis in gastric cancer cells while Ala exhibited anti-proliferative activity against gastric and breast cancer cells in vitro. Similarly, Pro and Lys exerted anti-proliferative activity against prostate cancer cells [255]. This suggested that peptides with the presence of specific amino acids in their sequences could exert activity against different cancer cell metabolic pathways as observed for free amino acids. Indeed, several antiproliferative peptides have been isolated from proteins hydrolysates of marine microalgae and SPBs as summarised in Table 5. The anticancer activity of these peptides depends on their amino acid composition, sequence, length, and overall charge/hydrophobicity. High amount of hydrophobic amino acids can enhance interactions between anticancer peptides and the outer leaflets of tumour cell membrane bilayers, facilitating in selective and stronger cytotoxic activity against cancer cells [256,257]. In this context, pepsin has been considered as a most efficient enzyme in the production of anticancer peptides from proteins because pepsin preferentially hydrolyses peptide bonds of polypeptides containing hydrophobic amino acids, especially aromatic amino acid residues (phenylalanine, tryptophan, and tyrosine) [258]. Pepsin digestion of half-fin anchovy produced an antiproliferative peptide (Tyr-Ala-Leu-Pro-Ala-His, MW 670.4 Da) containing 50% of hydrophobic amino acids [259]. Highly hydrophobic low molecular weight peptides could exert higher anticancer activities, as these peptides have greater molecular mobility and diffusivity, providing better interaction with cancer cell components. Comparing anticancer properties of two peptide fractions of different MWs (5.0 and 3.6 kDa) purified from Alcalase hydrolysates of solitary tunicate (*Styela clava*) demonstrated that the 3.6-kDa fraction had higher anticancer activity against AGS, DLD-1, and HeLa cells [232]. Similar result was also observed for peptides produced from marine microalgae. Peptide fractions of <3 kDa obtained from *Dunaliella salina* by enzymatic hydrolysis decreased viability of SW480 cell by 50% at a concentration of 0.08 μM [260]. Anticancer properties of peptides may be attributed by the presence of charged (glutamic acid) and heterocyclic amino acid (proline) in their sequences. A peptide, BCP-A (Trp-Pro-Pro), enriched in proline isolated from blood of clam exerted strong cytotoxicity against PC-3, DU-145, H-1299, and HeLa cell lines and significantly changed the morphologies of the PC-3 cells [257]. Therefore, Otani and Suzuki [261] suggested that there might be a correlation between the positive charge strength of the peptides and the cytotoxic activity. Marine proteins typically enriched in charged and free amino acids are therefore ideal for efficacy mining and production of novel anticancer peptide-based drugs.

#### 3.2.5. Antimicrobial

Due to living in an aquatic environment, most marine organisms are constantly in direct contact with a diverse range of pathogenic microbes, often present in high densities of up to 10^6^ bacterial and 10^3^ fungal cells/mL. They host specific populations of microbes on their surfaces or within the confines of their tissues [269], and therefore develop different mechanisms to synthesize effective protective agents to survive under this aggressive microbial pressure [270]. Many antimicrobial peptides (AMPs) isolated from different marine species including microalgae and fish have been reported [270,271]. These AMPs are usually amphiphilic, cysteine-rich, and positively charged in their active forms [272,273]. Their structures have been shown to differ from their counterparts produced by terrestrial species. They have been classified into different families, i.e., defensin, parasin, cathelicidin and hepcidin, and piscidin and are typically species-specific, e.g., piscidin are derived from teleost fish [274]. Proteins, protein hydrolysates, and peptides from marine microalgae and fish have been reported to possess anti-microbial activity (Table 6). Gelatine extracted from black-barred halfbeak exerted inhibitory activity against Gram-positive *Micrococcus luteus* and *Bacillus cereus* at a concentration of 10 mg/mL with respective inhibitor diameter zones of 6.5–7.0 mm [275]. Hydrolysates produced from skin gelatine of this fish inhibited several bacteria strains including three Gram-negative (*Klebsiella pneumonia*, *Salmonella enterica*, and *Salmonella typhi*) and three Gram-positive (*M. luteus*, *Staphylococcus aureus*, and *B. cereus*) bacteria [275]. Antimicrobial activity of protein hydrolysates and peptides generated from skins of other fish were also reported [276]. Protein hydrolysate prepared from viscera of *Scorpaena notata* using a purified serine protease (Th-Protease) exhibited remarkable antibacterial activities in vitro. The purified peptide, FPIGMGHGSRPA, inhibited several pathogenic bacteria, such as *B. cereus*, *B. subtilis*, *S. aureus*, *Salmonella* sp., *Listeria innocua*, and *Escherichia coli* at IC_50_ concentrations of 2.0–3.8 μM [277]. These harmful bacteria were also inhibited by the AQ-1766 peptide extracted from marine microalgae, e.g., *Tetraselmis suecica* with minimal bactericidal concentrations (MBC) of 40–50 μM [278]. Similar bioactivities have also been reported for protein hydrolysates and peptides produced from other species such as *D. salina* [260] and *Chlorella pyrenoidosa* [279]. The peptide, Pa-MAP, isolated from the polar fish (*Pleuronectes americanus*) was recently in the spotlight due to its strong antiviral activity inhibiting 90% of HSV, compared to 97% by LL-37 (another known antiviral peptide) and 99% by antiviral drug acyclovir (ACV) [45].

## 4. Applications of Proteins and Protein-Based Products Recovered from SPBs and Marine Microalgae

### 4.1. Protein Concentrates and Protein-Derived Products

Proteins and protein derivatives play vital roles in the sensory and nutritional quality of food products, which are important for human nutrition and health. Awareness of such roles led to commercial applications of proteins and their derivatives in food and nutraceutical industries. Common applications include production of a variety of restructured and ready-to-eat food products [12,280,281]. Fish proteins have been used in many fish-restructured products such as crab and lobster meat analogues, fish portions, fish balls, fish burgers, and fish sausages [72]. Frankfurter-type fish sausages with a soft texture and light colour were produced from proteins recovered from cape hake [35]. Physio-chemical properties of paste products such as hardness, cohesiveness, and whiteness were improved by formulating with fish proteins isolated from different species [282]. As functional nutrients, proteins extracted from yellowfin tuna roes have commercial applications for the development of paste-based products. These proteins are also suggested candidates for development of protein-fortified and marine-infused products [27]. In this context, protein hydrolysates produced from green mussel was incorporated in gluten-free breads containing buckwheat flour, rice flour, and chickpea flour to enhance product flavour [29]. The nutritive value of cereal proteins, indicated by net protein utilisation, increased significantly from 50 to 67% after wheat flour was formulated with 3% fish proteins [283]. Similar significant results were obtained in several other formulated products, such as puffed corn snack [284], ice cream [284], bread [285], biscuits [286], and crackers [287], which highlight potential of fish proteins for the production of formulated products. Mayonnaise, produced by formulation with fish proteins used for production of extruded corn snacks, were accepted by children, whilst the nutritional quality of the snack improved at the same time [288]. Their supramolecular interactions facilitate the creation of different types of colloidal systems, which are used as carriers of fish and microalgal proteins to deliver nutrients, nutraceuticals, bioactive compounds, and drugs [38,39]. Due to repulsive forces fish protein reduces oil absorption. Fried fish cakes, prepared by coating refined fish myofibrillar proteins with a paste slurry, reduced oil in the fried products by 63% [289]. This highlights application of fish proteins in production of fried products with low oil content for improved health benefits.

### 4.2. Fish Collagen and Gelatines

Collagen, a structural protein which maintains the healthy structure of various tissues and organs, has a wide range of applications in food, nutraceutical, cosmeceutical, and pharmaceutical industries [290,291,292]. Due to religion and pathological risks of transferring some diseases from mammalian collagen and its derived gelatine, the products derived from SPBs have received increased commercial interest [293]. Compared to mammalian products, fish collagen and gelatine (FCG) have comparable properties, such as solubility, viscosity, and gel strength with various uses in multiples industries [68,294]. Making use of their gelation ability and functional properties, SPB-derived collagens have been used in production of sausages to improve their rheological properties and to produce edible sausage cases. SPB-derived collagen films function as water and oxygen barriers that also prevent the migration of moisture and thereby inhibit undesirable biochemical changes, providing efficiently strategic protection for food products. Meat products coated with SPB-derived collagen films showed an extended shelf life, reduced purge (fluid loss), aroma and colour deterioration, and spoilage, while simultaneously improving sensory scores [295]. FCG act as emulsifiers, foaming agents, colloid stabilizers in food and beverage products [290,296,297]. Due to their synergetic benefits of improved functional properties and enhanced nutritive quality with associated health benefits [298], FCG have found applications in the production of health food products. As humans age, collagen synthesis decreases, causing tissues to become thinner, weaker, and less supple. Supplementing diets with sufficient collagen is considered to be the best solution, as it helps to uphold skin, hair, nails, and body tissue strengths [290]. Thus, a variety of food and beverage products incorporating FCGs have been developed, such as confectionery [295], dietary supplements [299,300], and functional foods. Drinks containing FCG are trendy products in the global market [290,298], due to functions in skin repair and tissue regeneration, and are therefore used in the development of cosmeceutical products [301]. Jellyfish collagen and its hydrolysed products alleviated UV-induced abnormal changes of the antioxidant defence systems that protects skin lipid and collagen [302]. Due to their excellent biocompatibility, biodegradability, high cell adhesion properties, and weak antigenicity, different types of functional biomaterials such as gels, scaffolds, sponges, films, membranes, and composites prepared from SPB-derived collagen have been used in medical and pharmaceutical industries [303,304]. They have been used as efficient drug carriers for cancer treatment or agents promoting cartilage and bone formation [41]. Wound healing and prophylactic treatment of bone and soft tissue infections are the main clinical applications of SPB-derived collagen [293]. Collagen has a role in the formation of tissues and organs [303,304], while it also contributes to the promotion of cell growth and differentiation and the regulation of various cell functions [305]. FCG-based biomaterials are less stable than those obtained from terrestrial mammals, but modifications through mixing with neutral buffer (1-ethyl-3-(3-dimethylaminopropyl)-carbodiimide) or hybrid-FCG through lyophilization with chitosan/hydroxyapatite significantly improved their stability [291].

### 4.3. Bioactive Peptides and Active Amino Acids

Microalgal and fish bioactive peptides including those derived from viscera, blood, collagen, and gelatine have demonstrated potential applications as functional and active ingredients in food, nutraceutical-, cosmeceutical-, and pharmaceutical products. Antioxidant and antimicrobial peptides derived from microalgae and SPBs can be used in food products as natural preservatives [306]. Incorporation of these bioactive peptides into packaging materials or films prepared from gelatine can extend the shelf-life of food products [307]. Fish gelatine films that incorporate lysozyme inhibit the growth of Gram-positive bacteria (*B. subtilis* and *S. cremoris*) at very low concentrations (0.001%), while such films incorporating a combination of lysozyme and catechin had antimicrobial activity against both Gram-positive and Gram-negative bacteria [308]. Gelatine hydrolysates have been used as plasticizers to improve flexibility and permeability of functional films prepared from fish myofibrillar proteins [309]. Protein hydrolysates and peptides produced from fish such as blue whiting (*Micromesistius poutassou*) with antioxidant activity had useful applications as nutraceuticals in beverages [310]. Many fish bioactive protein hydrolysates and peptides used for production of Food for Specified Health Use (FOSHU) have been summarised, which have been commercialised with several health beneficial claims such as anti-hypertension (e.g., Lapis Supporta, Valtyron, and PeptACE), relaxing (Stabiliuma 200, Protizena, and AntiStress 24), lowered glycaemic index (Nutripeptin), improving gastrointestinal health (Seacure), antioxidant, lowered glycaemic index, and anti-stress (Fortidium Liquamen) [211]. These products are available as beverages, jelly, powdered soup, and dietary supplements [311]. Peptides with antioxidant, anti-inflammatory, melanin synthesis reducing, inhibition of tyrosinase and matrix metalloproteinase bioactivities are important ingredients in cosmeceuticals, as they protect skin against aging and wrinkling [301]. Biopeptides that stimulate skin protein synthesis and deliver important cofactors for healing find applications in the treatment of skin diseases [312]. Fish and microalgal peptides and protein hydrolysates with photo-protective and anti-photoaging activities provide prophylactic treatments for the prevention of solar-induced skin damage [301,313]. Such bioactivities were observed in collagen hydrolysates and gelatine peptides of jellyfish [302], cod skin [314], Pacific cod [211], salmon [315], and tilapia [316]. Moreover, compared to animal collagen, SPB-derived products are better absorbed [317], exhibit improved mechanical strength, and low odour [318]. In addition, since collagen peptides support bone, joint, muscle, skin, and even ligament and tendon integrity, collagen hydrolysates have pharmaceutical applications in the treatment of osteoarthritis and other joint disorders [319].

### 4.4. Enzymes

Enzymes with their catalytic power and therapeutic effects have become an indispensable part of several industrial productions and medical treatments. They can efficiently catalyse numerous biochemical reactions in production of many commercial products such as detergents, food, flavours, agrochemicals, cosmetics, and pharmaceuticals or are capable to prevent and treat certain diseases as direct drugs [113,320,321]. Therefore, the global demand for enzymes is constantly increasing and predicted to surpass USD10 billion by 2024 [145]. Marine biomass, including SPBs and microalgae, has been identified as vital sources of different enzymes for various applications [141,322]. Of these applications, the use of SPB-derived enzymes for seafood processing is considered economically feasible because of low cost due to abundant availability at processing plants. In fact, these enzymes have been used in several seafood processes such as fermentation and curing of fish, hydrolysed product production, and extraction of bioproducts. SPB-derived enzymes are used to treat wastewater (stickwater) generated at seafood processing plants to reduce their viscosity [113]. Due to fundamental differences in biochemical compositions of skin and muscle, some enzymes have the capability of removing only the outer layer of muscles without damaging the original muscle tissue, which leads to useful applications such as purification of fish roes, descaling of fish skins, and removal of undesirable parts (skins, inedible portions, membranes, tissues, and organs) [113,323]. Another application is in dairy production, removing oxidized flavour from milk. Fish chymosin has been used as a protein coagulant for cheese production. The capability of SPB-derived enzymes to catalyse reactions at low temperature and high pressure are considered important characteristics determining competitiveness in industrial applications. Since the use of enzymes in industrial processes adds enzyme cost and costs of maintaining enzyme-specific conditions during processing, enzymes with high activity at low concentrations and mild conditions (temperature and pH) are of great interest. Species diversity of fish and microalgae combined with adaptations to various extreme conditions in marine environments are promising sources to provide enzymes which flexibly catalyse processes under a variety of different processing conditions. For example, pepsin and pepsin-like enzymes isolated from the stomach of cold-water fish (Pectoral rattail) and trypsin derived from Atlantic cod maintain high catalytic rates under high-pressure and low temperatures [324]. Other promising applications of SPB-derived and microalgal enzymes are in the cosmeceutical, nutraceutical, and pharmaceutical industries. SPB-derived enzymes that selectively degrade tissues have been used for production of skin-peeling agents in the cosmetic industry. An enzyme extracted from the hatching fluid of salmon can gently remove the outer dead layer of human skin without destroying the skin itself and is now produced as an exfoliating and skin rejuvenation product known as Zonase X™ [321]. Production of nutraceuticals such as the long-chain polyunsaturated fatty acids (PUFAs) omega-3, such as DHA, and EPA can be achieved with lipases recovered from SPBs and microalgae. Enzymes derived from these sources can also be used for the biosynthesis of biopeptides [325] and other active compounds, including polyketides, carotenoids or oxylipins [145]. Particularly, the enzyme L-asparaginase, present in various microalgae species [326,327], can be used for the treatment of acute lymphoblastic leukaemia, acute myeloid leukaemia, and non-Hodgkin’s lymphoma [328]. Similarly, superoxide dismutase (SOD) and metalloenzymes, use biomass contents are stimulated by environmental stress that leads to the activation of antioxidant defence mechanisms in microalgae, can be used to convert and neutralise superoxide radicals. Therefore, SOD has therapeutic and prophylactic applications in humans, as vaccination agents as an antigenic agent for the serodiagnosis of pathogens, and in the preservation of biological materials (organs for transplantation and sperm) [329]. Despite such potential, industrial production of SPB-derived and microalgal enzymes is still limited, although several enzymes produced from these sources are commercially available such as shrimp alkaline phosphatase, heat-labile Uracil-DNA glycosylase from Atlantic cod (https://arcticzymes.com), cold-active anti-bacterial chlamysin from a marine bivalve, lysozyme, and the cold-tolerant protease (commercially known as Penzim) (www.andra.is) from Atlantic cod.

## 5. Process Development for Production of Proteins and Protein-Based Products from SPBs and Marine Microalgae

### 5.1. Isoelectric Solubilisation/Precipitation (ISP) Process for Intact Protein Production

A common approach for protein extraction from different biomaterials including SPBs and microalgae is isoelectric solubilization and precipitation (ISP) known as pH shifting. In this technique, proteins of minced or blended materials are first dissolved in the extracting solvents by stirring or homogenising at optimal pH values for protein solubility. These pH values are in the acidic or alkaline range, but a neutral pH value is also used for extracting proteins from microalgae, i.e., 90–95% proteins of *Nannochloropsis oculata* are extracted at pH 7 [51]. Extracting proteins from this microalgal species at acid and alkaline conditions obtained under 20% (pH < 5) and 85–95% (pH 10) of protein [51], while these extracting conditions for SPBs such as herring (*Clupea harengus*) were 92% (pH 2.7) and 89% (pH 10.8) [330]. Both conditions are used in the extraction of collagens from SPBs. Alkaline extraction removes non-collagenous proteins, and the subsequent acidic extraction (preferably acetic acid) alters material structure and breaks down cross-links, facilitating subsequent solubilisation of collagens [293]. After extraction, insoluble residues are separated by centrifugation or filtration. Proteins in the extracted solutions are then precipitated by adjusting the solution pH to an isoelectric point (pI) which varies depending on protein type, pH 5.2–6.0 for fish proteins [12] and 3.0–4.0 for proteins of different microalgae species [51,331,332]. Precipitating fish proteins, i.e., herring proteins, at pH 5.5 recovered approximately 96% and 94% for acidic and alkaline extractions respectively [330], but 95–97% of microalgal proteins were precipitated at pH 3.0 for neutral and alkaline extracts [51]. Soluble proteins (3–4%) in wastewater generated from surimi processing and from squid chitin production were also recovered using precipitation at pH 3.5 [333,334]. Protein precipitation could be enhanced by co-precipitation with coagulants or flocculants which are adsorbed on the adjacent protein surfaces to bind them or to reduce the potential repulsion energy between adjacent proteins due to opposite charge to the suspended proteins [37,335]. Precipitation improvements with chitosan, alginates, and chitosan–alginate complexes were reported for the recovery of proteins from different SPBs, like fish [336], mussels [337], shrimp [338], and lobster [19]. Protein degradation may occur during extraction due to the presence of proteolytic enzymes, but this can be controlled by extraction at low temperature (4 °C). Extracting proteins from fresh raw SPBs is preferable compared to extraction of frozen material for production of functional and nutritional proteins. Recovered protein gels from alkaline pH-shift extractions had higher breaking strength and deformation stability than those from acidic ones [339]. Proteins extracted from microalgae, i.e., *Haematococcus pluvialis*, at acidic and neutral conditions, showed comparable emulsification, but emulsion stability and protein recovery yields of neutral extractions were higher than for acidic ones (94 and 73% vs. 84 and 64%, respectively) [340]. Proteins extracted at pH 6.5 from *Tetraselmis suecica* exhibited superior surface activity and gelation behaviour compared to whey protein isolates [341].

Thus ISP is a suitable method for recovery proteins from SPBs and microalgae and has advantages, such as the process being simple and scalable to pilot and industrial scales for different SPBs and microalgae [342,343]. Since the process can be conducted under mild conditions (4 °C, pH 6.5–11.5), recovered proteins are if high quality and can be used for the production of functional food [344], functional ingredients [345] or nutraceutical components [344]. Recovered fish and microalgal proteins through ISP displayed several functionalities [51,346], such as high oil/water binding and excellent film and gel forming capacities [347], solubility and foam stability [347], and stable emulsions [348]. Due to the selectively of the process, extracted proteins contain a low lipid content, offering a significant nutritional advantage as lipid oxidation is largely avoided [346]. Furthermore, presence of bio-toxic compounds, such as dioxin and polychlorinated biphenyls (PCBs), is significantly reduced by ISP [349] and reported toxic metal contamination is also very low compared to those using conventional extraction [19]. The above highlights that ISP extraction offers significant advantages for protein recovery from underutilised bioresources.

### 5.2. Hydrolysis Processes for Production of Protein Hydrolysates, Peptides, and Amino Acids

Proteins and protein complexes in SPBs and microalgae can also be hydrolysed into polypeptides, peptides, and amino acids via chemical (acids or alkalis) or enzymatic reactions (fermentation or enzymatic hydrolysis).

#### 5.2.1. Chemical Hydrolysis (Using Acids or Alkaline Extraction of Protein Hydrolysates, Biopeptides, Collagen, Gelatine, and Enzymes from SPBs and Microalgae)

Chemical hydrolysis cleaves protein peptide bonds and either acids or alkalis can be used. Acidic hydrolysis is more commonly used for fish protein recovery [350]. In acid hydrolysis, SPBs proteins are typically reacted with hydrochloric or sulfuric acid at high temperature and high pressure (128–131 °C, 220–310 MPa) [351]. The hydrolysate is then adjusted to pH 6.0–7.0, before it is concentrated or dried to obtain a protein paste or powder. Despite producing protein hydrolysates with good solubility, high content of salt generated by neutralisation combined with loss of nutrients such as essential amino acids (i.e., tryptophan) during hydrolysis render the products inappropriate for food applications. Similarly, hydrolysis of SPBs using alkali reactants (primarily sodium hydroxide) produces hydrolysed products with poor functionality and nutritional quality. This is indicated by the loss of nutrients (cysteine, serine, and threonine) via β-elimination reactions and formation of several undesirable substances (lysino-alanine, ornithine-alanine, lanthionine, and β-amino alanine) in the products [32]. In addition, L-amino acids are transformed into d-amino acids which are not absorbed by humans [352]. With such limitations of acidic and alkaline hydrolysates on quality, hydrolysed products derived from chemical hydrolysis are suitable for production of fertilisers [32,353]. Despite, chemical hydrolysis is still being used in industry for production of commercial peptides and protein hydrolysate complexes containing aromatic compounds from SPBs because the process is simple and inexpensive [64].

#### 5.2.2. Autolytic Hydrolysis (Fermentation)

Protein recovery from SPBs can be conducted by an autolytic process defined as fermentation in which fish proteins are hydrolysed by the action of the proteolytic enzymes of the fish itself. Fish viscera and digestive tracts are primary sources of digestive enzymes such as the serine proteases, trypsin, and chymotrypsin, and the thiol protease pepsin while fish muscle cells contain lysosomal proteases or catheptic enzymes [32]. These enzymes all participate in the fermentation process to some degree. The resulting relatively viscous liquid is enriched with small peptides and free amino acids. Due to the complex enzyme mixture and because different enzymes have different requirements for their activation/activity, the end products can differ significantly in their molecular profiles, depending on conditions used. Moreover, season, gender, age, and species influence the endogenous enzyme profiles and concentrations present, which results in the process being difficult to control and attain hydrolysates with specific molecular properties [32]. Despite identified quality limitations, fermentation with endogenous proteolytic enzymes is used for production of fish sauce and fish silage from underutilised SPBs.

Fish sauce is a fermented fish product consumed worldwide with a long history of production. Underutilised fish from one or more species are immersed in a solution of 20–40% salt and fermented at ambient tropical temperatures. The visceral proteolytic enzymes work together with catheptic enzymes to hydrolyse fish proteins. Since the process naturally occurs at a neutral pH with no base or acid supplementation, the contribution of acid-dependent proteolytic enzymes such as pepsin to the hydrolysis process is limited [32]. Additionally, at a high salt concentration and under anaerobic conditions (conditions inhibit the growth of spoilage micro-organisms) endogenous serine proteases transform fish muscle into a liquefied fish sauce with up to 50% nitrogen recovery (peptides and amino acids) after 6–12 months [354]. Lower salt concentrations, however, improve yields, lower the levels of volatile acids, and better-balance amino acid composition [355]. Although products produced from fish sauce and fish silage share several similar characteristics, the process is different. In fish silage, underutilised fish sources are mixed with strong mineral acids or organic acids to acidify the fish substrate. At acidic conditions (pH < 4), the serine proteases are generally inactive while pepsin and the catheptic enzymes present in high contents in fish visceral and muscles are highly active, producing a slurry containing up to 12% peptides and amino acids after several weeks. The processing time primarily depends on the visceral ratio and temperature. If it is fermented at 23–30 °C, up to 80% of fish silage proteins solubilise after 1 week [356]. The use of lactic acid bacteria as hydrolysis agents in the fish silage production has been reported as an advantage because the bacteria produces acid to lower pH and inhibit competing spoilage bacteria [357]. Fish silage is primarily used as feed for young animals due to the extensive hydrolysis of the proteins. To succeed in incorporation for animal feed production, fish silage proteins must be intact proteins or peptides rather than free amino acids, which have lower absorbability [358]. The shortening of processing time and addition of commercial proteases may be useful for production of fish silage with such composition. In fact, hydrolysates obtained from hake autolysis for 24 h at optimal conditions for the native enzymes (50 °C and pH 7.0) had protein efficiency ratios essentially equivalent to that of casein [359]. Protein hydrolysates prepared from shrimp heads (*Penaens vannamei*) at pH 7.85 and 50 °C for 3 h were enriched in essential and savoury amino acids, and have been proposed as functional food ingredients or flavour enhancers [154]. The development of a bitter taste of the silage limits incorporation levels in animal feeds and renders products unpalatable for humans. Due to the process being simple, no enzyme costs, and resulting hydrolysates containing functional and bioactive peptides, production of fish silage from SPBs has potential applications in nutraceutical and pharmaceutical industries [360].

#### 5.2.3. Enzymatic Hydrolysis

Hydrolysis with selected proteolytic enzymes has been used as a controllable strategy for converting SPBs and microalgae into marketable products, as it allows control of the degree of protein cleavage in the substrate. Hydrolysates with desired molecular structures, functional properties, and bioactive functions can be produced using appropriate enzyme/substrate ratios and reaction times [361]. Typically, biomaterials such as SPBs are first ground and then mixed with an appropriate amount of water to protein concentration between 8% and 12% [362]. Temperature (generally 35–65 °C) and pH of the substrate are adjusted to the optimal conditions for the hydrolysing enzyme, i.e., pH 2 for acidic (pepsin), pH 7 for neutral enzymes (papain, bromelain, neutrase, and flavourzyme), and pH 8.5 for alkaline enzymes (alcalase). Enzyme or enzyme mixtures are shaken, stirred or blended for a certain time period [350] and the process is terminated by heating to 75–100 °C for 5–30 min or by changing the pH to inactivate the enzyme [32]. After separation of insoluble residues, the hydrolysed liquid is concentrated or dried to obtain a protein paste or powder. A wide variety of commercial enzymes can be used for protein recovery from fish and microalgae, but the choice is typically determined by a combination of efficacy and economics [352]. Pronase, pepsin, papain, and pancreatin are promising enzymes for protein recovery due to high activity per unit weight [32], while pepsin has been reported to obtain a high level of solubilization [352] and to limit microbial growth to the acidic nature of the process, but hydrolysates are characterised by poor functionality due to excessive hydrolysis [92]. Thus, neutral and slightly alkaline enzymes have recently been used more often. Alcalase, an alkaline enzyme is one of the best choices for producing functional fish protein hydrolysates [363]. Alcalase hydrolysis of capelin had superior protein recovery compared to the alkaline proteases neutrase and papain and achieved lowest lipid contents (0.18%). Compared to Flavourzyme 1000 L, Corolase PN-L and 7089 for fish protein recovery from salmon, use of alcalase was more cost-effective [364]. Enzymatic hydrolysis yields higher protein recovery compared to the autolytic process. For example, three times higher protein recovery (70.6%) was obtained from ground capelin (*Mallotus villosus*) using alcalase compared to 22.9% achieved with hydrolysis by endogenous enzymes [363]. Since enzyme-catalysed hydrolysis produces protein hydrolysates with higher functional and nutritional quality compared to chemical or autolytic hydrolysis, this process has been used for protein recovery from different SPBs such as finfish (tuna [227], salmon [195], flounder [198], pollack [42]), crustaceans (crab [365], krill [186], lobster [18]), molluscs (blue mussel [82,366]), coelenterates and echinoderms (jellyfish, sea cucumber [367,368]) and microalgae [177]. This process produces bioactive peptides with applications in the food, nutraceutical, cosmetical, and pharmaceutical industries (Table 2, Table 3, Table 4, Table 5 and Table 6). Despite the promise, low yields, enzyme costs, and bitter taste of protein hydrolysates still pose limitations to adoption of the process [32], which can be overcome by using intensified processes. Microwave- [18], ultrasound- [369] or high hydrostatic pressure-intensified [370] enzymatic hydrolyses improved yields and enzyme costs could be reduced through immobilisation [371]. The bitter taste of protein hydrolysates from herring was avoided by using a mixture papain and bromelain [350]. Thus recent progress made takes enzymatic hydrolysis for protein recovery from SPBs and microalgae a step closer to commercial reality.

### 5.3. Economic Feasibility and Industrial Production of Protein-Based Products from Underutilised Marine Bioresources

Although utilisation of underutilised bioresources for production of proteins and protein-based products has been demonstrated to be promising at laboratory scales, translation to industrial production requires conducting these processes at pilot and plant scales. This can be achieved by either simulation using different mathematical models or by conducting actual trials [372]. To significantly save time and cost for process development, simulation has been used to analyse technical and economic feasibility of industrial production of proteins from different SPBs (kingfish [373], catfish [374], tuna [375], and shrimp [376]) and microalgae [377,378]). Industrial production of fish protein hydrolysate (FPH) from yellow tail kingfish processing by-products (YTKPBs) by chemical and enzymatic hydrolysis with microwave intensification was simulated using a commercial simulator, SuperPro Designer [373]. An annual feeding scale of 3900 tonnes, equivalent to a maximum quantity of YTKPBs annually produced in South Australia was set as the basis. Economic outcomes were sensitive to cost of YTKPBs and the selling price of FPH. The simulation showed that both production processes were economically viable, if the cost of YTKPBs ranged from 1 to 3 USD/kg and the selling price of products was 20–40 USD/kg, achieving a payback on investment of 2 years. Microwave-intensified chemical hydrolysis was, however, more profitable than microwave-intensified enzymatic hydrolysis [373]. Based on modelled feasibility, one industrial plant (SAMPI) with a designed capacity of 3000 tonnes/year was built in Port Lincoln, South Australia to produce commercial fish protein hydrolysate from tuna and kingfish processing by-products using enzymatic hydrolysis [379]. Similarly, industrial production of fish protein concentrate and crude enzymes was also shown to be economically feasible at industrial scale for catfish processing by-products [374], gelatine from tuna [375], shrimp paste [376], and microalgal proteins [378]. Enzymatic hydrolysis for production of collagen hydrolysate from codfish skin was successfully scaled up from a laboratory scale to pilot and plant scales with capacity of 100 and 2000 L, respectively [380]. Production yields at pilot and plant scales were >66%. Importantly, the quality of products obtained in large-scale production was similar to those achieved at laboratory scale (~95% of oligopeptides), with peptides of 3 kDa and <1 kDa accounting for 95% and 60% of the yield, respectively. The product demonstrated potential applications in biomedical, functional food, pharmaceutical, and cosmetic industries because it was nontoxic and non-irritating to skin and showed good moisture-retention, antioxidant activity, and promoted cell viability in tests with human dermal fibroblasts.

Despite gaining some successful translations, industrial production of commercial proteins and protein-based products from SPBs and marine microalgae are still facing with several challenges though. One technical challenge is a lack of scalable and consistent methods which could be applied to a variety of feedstock. For example, mechanical and physical disruptions of cell walls for protein extraction from microalgae are still economically challenging due to energy demand [54,381]. The use of some technologies such as centrifugation, freeze-drying, and chromatographic fractionation used for separation, dehydration, and purification in production of protein powder, biopeptides and enzymes are still not cost-effective at industrial scales for some feedstock [350,382]. Utilisation of SPBs also poses some logistical issues. Utilisation of SBPs for producing commercial products requires implementation of appropriate handling procedures from harvesting to processing. They need to be transported quickly to processing units to minimise unnecessary biochemical changes and avoid spoilage [350]. Appropriate handling for food safety, such as cold-chain management, are, however, hardly applied at the SBP-production site due to lack of well-designed models for collection, storage, and transportation [383]. To overcome this major limitation, close collaboration between fish processing plants and by-product utilisation facilities is required. Ideally, a cluster of several processing units should be set up on a shared site to improve transportation of SPBs among units [350]. Moreover, typically utilisation of SPBs for production of protein-based products and fishmeal are competitive and mutually exclusive approaches. Currently 30% of SPBs are used for production of fishmeal and oils, but this figure is set to increase due to higher profitability, as a result of increasing in selling price and global demand of fish meal and fish oils [11,384].

### 5.4. Ultrasound and Supercritical Carbon Dioxide (SCrCO_2_) as Promising Extraction and Separation Technologies for Protein Recovery from Marine Bioresources

Ultrasound has been widely used to intensify many extraction processes, due to cavitation induced by the treatment which disrupts biomass. The quick formation and sudden and violent collapse of cavitation bubbles leads to turbulence and shearing in the cavitation zone [385]. Such synergic effects disrupt cell walls and membranes, creating micro-cavities in the tissues which improves solvent penetration, resulting in the transformation of material structure and improved mass transfers, which in turn reduce processing time, solvent use and increase yields [386,387]. A 15.6% increase in extracted protein yields from tilapia was achieved with ultrasound-intensified ISP under alkaline condition [388]. Similarly, the protein yields of >95% [389,390] and 99% were achieved with ultrasonic extraction under alkaline conditions (pH 13) from mackerel by-products and lobster heads, respectively [19]. Moreover, 1.5–2 times higher extraction yields and 10-fold lower consumption of acetic acid compared to conventional processes were achieved for ultrasound-intensified production of collagen from skins of flatfish (*Paralichthys olivaceus*) [342]. Ultrasound-intensified hydrolysis also improved extraction yields, reduced extraction times for collagen extraction from the skin of Japanese sea bass (*Lateolabrax japonicus*) [391] and microalgae [392]. Ultrasonic extraction yielded relatively intact proteins, suggesting that little protein degradation occurs. In fact, characterisation of the ultrasound-extracted collagen showed that the major components of the collagen including the α1, α2, and β chains were unchanged. Similarly ultrasound-intensified pepsin hydrolysis yielded improved efficiency of extraction of natural high-quality collagen [369] with an intact triple helix structure, which was validated by circular dichroism analysis, atomic force microscopy and Fourier-transformed infra-red spectroscopy (FTIR) [393]. In addition, digestibility and nutritional value of ISP-recovered proteins was higher than for conventional extracts and contamination with toxic heavy metals was significantly lower [19]. Hence, ultrasound is promising technology for the intensified production of proteins from SPBs and microalgae.

Separating proteins and their derivatives from the solution is a vital step in protein recovery and production. Several different techniques could be applied [394], but isoelectric precipitation is most commonly applied due to significant advantages as discussed in Section 5.1. The use of inorganic acids, such as HCl or H_2_SO_4_ to denature proteins, however, requires costly subsequent neutralisation, dialysis, and effluent treatment, and the final products may be contaminated with chemical residues. The use of volatile electrolytes such as CO_2_, as an alternative to the use of acids in protein precipitation, overcomes these disadvantages and adds additional benefits to the process, such as preventing protein denaturation caused by localised pH extremes and greatly reduced saline effluent generation. CO_2_ can be easily separated by pressure release and economically recycled for multiple precipitation cycles [395]. The mild acidic and anti-solvent properties of the liquid and supercritical CO_2_ have been shown to aid the separation and purification of food proteins. Strong interactions with various proteins in both aqueous [396,397] and organic solutions [398] of SCrCO_2_ has been demonstrated, triggering the formation of protein particles and/or the precipitation of selected proteins via acidification or anti-solvent effects. These properties have been utilized to extract, isolate, or purify diverse types of proteins from different sources such as soybeans [399,400,401], milk [402,403], fish [404,405], fruits [406], porcine insulin [395], or model proteins [407]. The solubility of SCrCO_2_ in protein solutions and the diverse effects of SCrCO_2_ on the protein structure and pH have been studied [408,409,410,411]. SCrCO_2_ lowers the pH of protein solutions to generally below pH 5 and precipitates soluble proteins at temperatures of around 35–40 °C [412,413,414]. Once SCrCO_2_ is mixed and dissolved in water, formation of carbonic acid results, leading to the decreased pH of the protein solutions (as low as pH 4.5). The final pH can be controlled by the solubility of the CO_2_ gas, which depends on thermodynamic equilibrium of SCrCO_2_ and pressure [415]. Food proteins, such as α-lactoalbumin, can be selectively precipitated at temperatures of 60–65 °C and a pH between 4.2 and 5.0 [416,417]. SCrCO_2_ precipitation of proteins can be intensified using a co-solvents such as ethanol [398]. Operation in continuous mode is a significant advantage of the process [403] which typically yields peptides with increased functionalities [418]. Integration of ultrasound-intensification into the SCrCO_2_ extraction process may provide a novel strategy to further improve yields, functionality and solvent use setting the stage for green and sustainable recovery of proteins from underutilised marine bioresources.

## 6. Conclusions

A large increase in global demand for protein has driven production from hitherto underutilised resources. SPBs and marine microalgae are two advantageous feedstock offering multiple technical advantages for production due to their abundant availability, low material cost and large yields/production potential. Various types of SPBs are produced annually are being discarded, incurring financial and environmental costs. This review demonstrates that efficient utilisation for protein recovery is suitable to fill the gap in protein demand. It is concluded that SPB-derived proteins and protein hydrolysates are highly nutritional, making them suitable for food production. Furthermore, it is shown that collagen, gelatines, bioactive peptides, enzymes, and enzyme inhibitors possess several useful bioactivities with potential applications in nutraceutical, cosmeceutical, and pharmaceutical industries. Currently, ISP and enzymatic hydrolysis are feasible technologies for protein recovery from SPBs due to being simple applications, industrial scalability, and adequate preservation of nutritional quality and bioactivities. However, based on the review, it is concluded that technologies could be improved further, if they were integrated with ultrasonic intensified extractions and supercritical carbon dioxide for separation, which would yield greener and even more sustainable production. The review highlights that processes are economically and environmentally achievable at industrial scales, demonstrating that the approach offers a sound platform for sustainable development of several industries, yet some critical hurdles such as adequate integration of processes for improved techno-economic outcomes and product quality and potential are yet to be fully addressed at industrial scales.

## Figures and Tables

**Table 1:** Seafood processing by-products (SPBs) generated from common marine animal species, their ratios, protein contents, and potential for recovery of proteins and protein-based products.

Marine Groups	Typical Species	By-Product Types	Ratio of By-Products (%) of Total Weight	Protein Contents (%)	Type of Proteins or Protein-Derived Products	References
Finfish	Pollock, cod, hake, haddock, salmon, tuna, herring, mackerel, and among many others	Heads	15–20	11.9–12.9 ^a^	Proteins, protein hydrolysates, biopeptides	[63,64,65,66]
		Frames	10–15	11.5–17.5 ^a^	Collagen, gelatine, protein hydrolysates, biopeptides	[63,65,67]
		Skins and fins	1–3	24.8–27.0 ^a^	Collagen, gelatine, protein hydrolysates, biopeptides	[63,65,68]
		Bones	9–15	36.3–56.8 ^b^	Collagen, gelatine, protein hydrolysates, biopeptides	[69,70]
		Scales	3–5	41–81 ^b^	Ichthylepidin and collagen, biopeptides	[69,71]
		Viscera (livers, roes, and milts)	15–20	12.9–14.8 ^a^	Enzymes, protein hydrolysates, peptides, biopeptides	[63,65,66,67,72]
		Blood	2–7	0.8–5.7 ^a^	Plasma proteins, active amino acids, enzyme inhibitors	[73,74]
Crustacean	Krill, shrimp, crap, crayfish, lobster	Shells, tails	15	29–40 ^b^	Shell proteins, caroteno-proteins	[18,75,76]
		Heads	25	43.5–54.4 ^b^	Shell and meat proteins	[19,76,77]
		Viscera (livers, roes)	5	41 ^b^	Enzymes, protein hydrolysates, peptides, biopeptides	[60,78]
Mollusc	Oyster, mussel, clam, scallop	Shells	75–80	1–5 ^b^	Bioactive peptides	[79,80,81,82]
		Body parts and organs		58.7 ^b^	Enzyme, protein hydrolysate, biopeptide, flavour	[82,83]
	Cuttlefish, Squid, Octopus	Ink bags, organs, and non-edible portions	25–44.3	5–22 ^a^	Enzymes, bioactive peptides, food flavours, taurine	[84,85,86,87]
Coelenterate and echinoderm	Sea urchin	Shells, viscera	40.7–77.9	4.1–5.0 ^b^	Bioactive proteins for self-assembly of skeletal structure	[88]
	Sea cucumber	viscera		4.5 ^a^	Enzymes, protein hydrolysate, bioactive peptides	[89]
	Jelly fish			3–7 ^a^	Protein hydrolysate, bioactive peptides, collagen, gelatine	[83,90]

^a^ Wet weight basis. ^b^ Dry weight basis.

**Table 2:** Anti-hypertensive activity of peptides derived from SPBs and marine microalgae.

Anti-Hypertensive Assays	Peptide Names or Sequences	Efficiency (IC_50_, EC_50_) (μM)	Types of SPBs, Marine Species	Enzymes, Production Conditions	References
	GGPAGPAV GPVA PP GF	673.2 445.6 1912.4 178.1	Trimming of Atlantic salmon (*Salmo salar*)	Corolase PP	[195]
	Phe-Gly-Ala-Ser-Thr-Arg-Gly-Ala	14.7	Frames of Alaska pollock (*Theragra chalcogramma*)	Pepsin	[42]
	GDLGKTTTSNWSPP	11.3	Frame of bluefin tuna (*Thunnus thynnus*)	Pepsin	[168]
	-	Observed at 4.8 μM 85.8% ACE inhibition	Mince of Boarfish (*Capros aper*)	Protease AP	[196]
	EPLYV DPHI AER EQIDNLQ WDDME	118 48.7 420 270 31.6	Mince of Leatherjacket (*Meuschenia* sp.)	Papain Bromelain Flavourzyme 500 L	[197]
SBP6h (40 mg/kg)	MEVFVP VSQLTR	79 μM, 44.3 mmHg 105 μM, 34.3 mmHg	Mince of Olive flounder (*Paralichthys olivaceus*)	Pepsin	[198]
	-	3.9	Skin gelatine of Rockfish (*Sebastes hubbsi*)	Flavourzyme	[199]
	MVGSAPGVL LGPLGHQ	3.1 4.2	Skin gelatine of skate (*Okamejei kenojei*)	Alcalase 2.4 L	[200]
	-	0.4	Oysters (*Crassostrea gigas*)	Fermentation with 25% NaCl at 20 °C for 6 months	[201]
ACE inhibitor	-	1.6	Gelatine of giant squid (*Dosidicus gigas*)	Alcalase	[202]
Nitric oxide production No cytotoxicity on HUVECs	GMNNLTP (Gly-Met-Asn-Asn-Leu-Thr-Pro MW 728 Da) LEQ (Leu-Glu-Gln, MW 369 Da)	123–173	*Nannochloropsis oculata*	Pepsin, trypsin, αchymotrypsin, papain, alcalase, and neutrase	[177]
Spontaneously hypertensive rats (SHRs)	VEGY (Val–Glu–Gly–Tyr, MW 467.2 Da)	128.4	*Chlorella ellipsoidea*	Protamex, Kojizyme, Neutrase, Flavourzyme, Alcalase, trypsin, α-chymotrypsin, pepsin and papain	[203]
	WV (Trp-Val) VW (Val-Trp) IW (Ile-Trp) LW (Leu-Trp)	307.6 0.6 0.5 1.1	*Chlorella sorokiniana*	Protease N, pepsin, pancreatin	[204]
	GPDRPKFLGPF WYGPDRPKFL	5.73, EC_50_ 0.82, EC_50_	*Tetradesmus obliquus*	Alcalase	[205]
ACE-inhibitory	Peptides < 5 kDa	Observed at 4.8 μM 30.8–37.8%	*Chlorella sorokiniana*	Pepsin, bromelain, and thermolysin	[206]

**Table 3:** Antioxidative activity of peptides derived from SPBs and marine microalgae.

Antioxidant Assays	Peptide Names or Sequences	Efficiency (IC_50_, EC_50_, TE) (μM)	Types of SPBs, Marine Species	Enzymes, Production Conditions	References
ORAC	GGPAGPAV GPVA PP GF	5.5 9.5 12.5 19.7	Trimming of Atlantic salmon (*Salmo salar*)	Corolase PP	[195]
DPPH ABTS Hydroxyl	PAGT	25.8 EC_50_ 0.04 EC_50_ 4.3 EC_50_	Skin gelatine of Amur sturgeon (*Acipenser schrenckii*)	Alcalase 2.4 L	[226]
DPPH Hydroxyl Superoxide	APTBP	3.5 EC_50_ 1.0 EC_50_ 2.9 EC_50_	Backbone of bluefin tuna (*Thunnus thynnus*)	Pepsin	[227]
DPPH Hydroxyl Oxygen	FIGP	0.6 EC_50_ 0.4 EC_50_ 1.5 EC_50_	Skin of bluefin leatherjacket (*Navodon septentrionalis*)	Papain	[228]
DPPH	-	0.02 EC_50_	Half-fin anchovy (*Setipinna taty*)	Pepsin	[229]
DPPH Hydroxyl Alkyl Superoxide	-	Observed at 19.2 μM 45.8% 94.7% 64.8% 67.8%	Skin gelatin of rockfish (*Sebastes hubbsi*)	Flavourzyme	[199]
DPPH ABTS FRAP DPPH ABTS FRAP	-	6.8 μmol TE/g dw 65.5 μmol TE/g dw 2.6 μmol TE/g dw 6.3 μmol TE/g dw 59.4 μmol TE/g dw 2.7 μmol TE/g dw	Skin of seabass (*Lates calcarifer*)	Alcalase 2.4 L Protease from hepatopancreas of Pacific white shrimp	[230]
ABTS	EPGPVG LPGPAG LDGPVG EGPLG	1.25 μmol TE/g peptide 1.22 μmol TE/g peptide 1.36 μmol TE/g peptide 4.95 μmol TE/g peptide	Skin of unicorn leatherjacket (*Aluterus monoceros*)	Glycyl endopeptidase from papaya	[231]
DPPH ABTS		9.6 0.04	Shrimp (*Penaeus monodon* and *Penaeus indicus*)	Alcalase	
DPPH Oxygen radical 2,2-azino-bis(3-ethylbenzthiazoline)-6-Sulfonic acid cation		2.4 μM, IC_50_ 497.4 μmol TE/mg 48.4 μmol TE/mg 110.4 μmol TE/mg	Krill (*Euphausia superba*)	Pepsin	[186]
ABTS DPPH	F2 3.6 kDa	0.05 1.1	Solitary Tunicate (*Styela clava*)	Alcalase 2.4 L FG, Thermoase PC10F, pepsin	[232]
Hydroxyl Superoxide DPPH	Enzymatic hydrolysates	102−196 μg/mL	*Navicula incerta*	Alcalase, pronase-E, α-chymotrypsin, neutrase, papain, pepsin, and trypsin	[222]
	NIPP-1 (Pro-Gly-Trp-Asn-Gln-Trp-Phe-Leu) 1.171 kDa NIPP-2 (Val-Glu-Val-Leu-Pro-Pro-Ala-Glu-Leu) 1.108 kDa		*Navicula incerta*	Alcalase, α-chymotrypsin, neutrase, papain, pepsin, pronase-E and trypsin	[233]
ABTS DPPH	WPRGYFL (MW 937 Da) SDWDRF (MW 824 Da)	4.70, EC_50_ 14.0, EC_50_	*Tetradesmus obliquus*	Alcalase	[205]
Peroxyl DPPH Hydroxyl	LNGDVW	0.02 mM 0.92 mM 1.42 mM	*Chlorella ellipsoidea*	Papain, trypsin, pepsin and a-chymotrypsin	[234]
ORAC FRAP ORAC FRAP	-	14.0 μmol TE/g dw 478.9 μmol TE/g dw 15.0 μmol TE/g dw 155.7 μmol TE/g dw	*Porphyridium purpureum* *Phaeodactylum tricornutum*	Alcalase 2.4 L and Flavourzyme 500 L	[235]
Hydroxyl radical	MPGPLSPL (793.01 Da)		*Pavlova lutheri*	Proteolytic yeast Candidia rugopelliculosa	[236]
DPPH	Peptides < 5 kDa	Observed at 4.8 μM 46.9–50.9%	*Chlorella sorokiniana*	Pepsin, bromelain, and thermolysin	[206]

**Table 4:** Antidiabetic activity of peptides derived from SPBs and marine microalgae.

Antidiabetic Assays	Peptide Names or Sequences	Efficiency (IC_50_, EC_50_) (μM)	Types of SPBs, Marine Species	Enzymes, Production Conditions	References
	PGVGGPLGPIGPCYE CAYQWQRPVDRIR PACGGFWISGRPG	116.1 78.0 96.4	Longtail tuna (*Thunnus tonggol*)	Protease XXIII	[44]
	GGPAGPAV GPVA PP GF	8139.1 264.7 4343.5 1547.2	Trimming of Atlantic salmon (*Salmo salar*)	Corolase PP	[195]
	GPAE GPGA	49.6 41.9	Skin of Atlantic salmon (*Salmo salar*)	Flavourzyme	[250]
	AP VR	0.02 0.07	Skin collagen of Atlantic salmon (*Salmo salar*)	Alcalase 2.4 L, papain	[202]
	-	1.0% hydrolysate 122 pM CKK release	Mince of Blue whiting (*Micromesistius poutassou*)	Endopeptidase	[251]
	-	7.2	Mince of Blue whiting (*Micromesistius poutassou*)	Alcalase 2.4 L Flavourzyme 500 L	[252]
DPP-IV inhibitory	-	10.9 12.9	*Porphyridium purpureum* and *Phaeodactylum tricornutum*	Alcalase 2.4 L and Flavourzyme 500 L	[235]

**Table 5:** Anticancer activity of peptides derived from SPBs and marine microalgae.

Anticancer Assays	Peptide Names or Sequences	Efficiency (IC_50_, EC_50_) (μM)	Types of SPBs, Marine Species	Enzymes, Production Conditions	References
MCF-7	LPHVLTPEAGAT PTAEGGVYMVT	8.1 8.8	Dark muscle byproduct of longtail tuna (*Thunnus tonggol*)	Papain Protease XXIII	[262]
DU-145 cell	-	200	Half-fin anchovy (*Setipinna taty*)	Pepsin	[229]
Ca9-22	-	4.1	Roe of Rohu (*Labeo rohita*)	Protease N	[263]
MCF-7/6 MDAMB-231	Free amino acids, peptides with ~7 kDa	Exhibited cell growth inhibition	Blue whiting, cod, plaice, and salmon	Alcalase and protamex	[264]
Caco2 (Human colon) HepG2 (Human liver)	Fraction < 10 kDa Fraction 10–30 kDa Fraction > 30 kDa	Significantly inhibited the growth of both colon and liver cancer cells by 60%. <10 kDa fraction from shrimp shells (FL) inhibited growth of liver cancer cells alone by 55%, compared to controls	Shrimp shell	Cryotin enzyme	[265]
Female BALB/c mice with transplanted sarcoma S180 cells	MW < 3 kDa	Significantly inhibited the growth of transplanted sarcoma S180 cells in mice	Oyster (*Crassostrea gigas*)	Protease from *Bacillus* sp. SM98011	[266]
PC-3 DU-145 H-1299 HeLa	BCP-A (Trp-Pro-Pro), 398.4 Da	Cytotoxicity in a dose-dependent manner. Significantly changed the morphologies of the PC-3 cells. The percentage of early stage apoptotic PC-3 cells increased from 11.22 to 22.78% when they were treated with Trp-ProPro concentrations of 5 and 15 mg/mL, respectively, for 24 h.	Blood of clam (*Tegillarca granosa*)	Neutrase	[257]
PC-3 (prostate) A549 (lung) MDA-MB-231 (breast) Normal liver cells	Ala-Val-LeuVal-Asp-Lys-Gln-Cys-Pro-Asp	Lethal concentration (LC) 6.2, LC_50_ 6.5, LC_50_ 7.6, LC_50_ No cytotoxicity	*Ruditapes philippinarum*	α-Chymotrypsin	[267]
DU-145 cells	N Gln-Pro-Lys, MW 343.4 Da		Sepia Ink	Trypsin	[254]
MCF-7 (human breast carcinoma) U87 (glioma)		0.6 0.48	Gelatine of giant squid (*Dosidicus gigas*)	Esperase	[202]
AGS, DLD-1 HeLa	F2, 3.6 kDa	2.8 5.6 4.3	Solitary Tunicate (*Styela clava*)	Alcalase 2.4 L FG, Thermoase PC10F, pepsin	[232]
HepG2 cells	Polypeptide CPAP	426 μg/mL	*Chlorella pyrenoidosa*	Papain, trypsin, and alcalase	[268]
			*Navicula incerta*	Alcalase, α-chymotrypsin, neutrase, papain, pepsin, pronase-E and trypsin	[233]
SW480 (Colon cancer cell lines)	Peptides < 3 kDa	0.8	*Dunaliella salina*	Trypsin and chymotrypsin	[260]

**Table 6:** Antimicrobial activity of peptides derived from SPBs and marine microalgae.

Antimicrobial Assays	Peptide Names or Sequences	Efficiency (IC_50_, EC_50_) (μM)	Types of SPBs, Marine Species	Enzymes, Production Conditions	References
*Bacillus cereus* *Bacillus subtilis* *Staphylococcus aureus* *Salmonella* sp. *Listeria innocua* *Escherichia coli*	FPIGMGHGSRPA	2.9 2.0 2.4 3.5 2.4 3.8	Viscera of Small red scorpionfish (*Scorpaena notata*)	Crude enzyme from *Trichoderma harzianum*	[277]
Antibacterial	Gelatine (BG) Gelatine hydrolysate		Skin of black-barred halfbeak (*Hemiramphus far*)	Purafect	[275]
	AQ-3001, AQ-3002, AQ3369, AQ-3370, AQ-3371, and AQ-3372		*Tetraselmis suecica*	Acid extracts	[278]
*Escherichia coli* *Staphylococcus aureus*	Protein hydrolysate 63 kDa	59.4% 42.9%	*Dunaliella salina*	Trypsin and chymotrypsin	[260]
HSV-1		83 μg, EC_50_	Winter flounder (*Pleuronectes americanus*)	Synthetic peptide	[45]

## Abbreviations

ACE	Angiotensin-I-converting enzyme
DPP-IV	Dipeptidyl peptidase IV
FCG	Fish collagen and gelatine
FPH	Fish protein hydrolysate
ISP	Isoelectric solubilisation precipitation
MT	Million tonnes
MW	Molecular weight
PCs	Protein concentrates
PHs	Protein hydrolysates
SCrCO_2_	Supercritical carbon dioxide
SHR	Spontaneously hypertensive rats
SPB	Seafood processing by-products
YTKPBs	Yellow tail kingfish processing by-products

## References

[1] Henchion M., Hayes M., Mullen A.M., Fenelon M., Tiwari B. (2017). Future protein supply and demand: Strategies and factors influencing a sustainable equilibrium. Foods.

[2] Ritala A., Häkkinen S.T., Toivari M., Wiebe M. (2017). Single cell protein: State-of-the-art, industrial landscape and patents 2001–2016. Front. Microbiol..

[3] Daliri E.B.-M., Oh D.H., Lee B.H. (2017). Bioactive peptides. Foods.

[4] Chakrabarti S., Guha S., Majumder K. (2018). Food-derived bioactive peptides in human health: Challenges and opportunities. Nutrients.

[5] Morlighem J.-É.R., Radis-Baptista G. (2019). The place for enzymes and biologically active peptides from marine organisms for application in industrial and pharmaceutical biotechnology. Curr. Prot. Pept. Sci..

[6] Soto-Sierra L., Stoykova P., Nikolov Z.L. (2018). Extraction and fractionation of microalgae-based protein products. Algal Res..

[7] Shahidi F., Varatharajan V., Peng H., Senadheera R. (2019). Utilization of marine by-products for the recovery of value-added products. J. Food Bioact..

[8] OECD (2019). OECD-FAO Agricultural Outlook 2019–2028.

[9] Soldo B., Šimat V., Vlahović J., Skroza D., Ljubenkov I., Generalić Mekinić I. (2019). High Quality Oil Extracted from Sardine By-Products as an Alternative to Whole Sardines: Production and Refining. Eur. J. Lipid Sci. Technol..

[10] Karayannakidis P.D., Zotos A. (2016). Fish processing by-products as a potential source of gelatin: A review. J. Aquat. Food Prod. Technol..

[11] Šimat V., Vlahović J., Soldo B., Mekinić I.G., Čagalj M., Hamed I., Skroza D. (2020). Production and characterization of crude oils from seafood processing by-products. Food Biosci..

[12] Sasidharan A., Venugopal V. (2019). Proteins and co-products from seafood processing discards: Their recovery, functional properties and applications. Waste Biomass Valorization.

[13] Bendif E.M., Probert I., Schroeder D.C., de Vargas C. (2013). On the description of *Tisochrysis lutea* gen. nov. sp. nov. and *Isochrysis nuda* sp. nov. in the Isochrysidales, and the transfer of *Dicrateria* to the Prymnesiales (Haptophyta). J. Appl. Phycol..

[14] Aime F., Concepcion H. (1985). Marine microalgae as a potential source of single cell protein. Appl. Microbiol. Biotechnol..

[15] Chen Y., Wang C., Xu C. (2020). Nutritional evaluation of two marine microalgae as feedstock for aquafeed. Aquac. Res..

[16] Benedetti M., Vecchi V., Barera S., Dall’Osto L. (2018). Biomass from microalgae: The potential of domestication towards sustainable biofactories. Microb. Cell Factories.

[17] Sitepu E.K., Heimann K., Raston C.L., Zhang W. (2020). Critical evaluation of process parameters for direct biodiesel production from diverse feedstock. Renew. Sustain. Energy Rev..

[18] Nguyen T.T., Zhang W., Barber R.A., Su P., He S. (2016). Microwave-intensified enzymatic deproteinization of Australian rock lobster shells *(Jasus edwardsii)* for the efficient recovery of protein hydrolysate as food functional nutrients. Food Bioprocess. Technol..

[19] Nguyen T.T., Luo X., Su P., Balakrishnan B., Zhang W. (2020). Highly efficient recovery of nutritional proteins from Australian Rock Lobster heads (*Jasus edwardsii*) by integrating ultrasonic extraction and chitosan co-precipitation. Innov. Food Sci. Emerg. Technol..

[20] Gates K., Venugopal V. (2009). Marine products for healthcare: Functional and bioactive nutraceutical compounds from the ocean. Functional Foods and Nutraceuticals Series.

[21] Korhonen H., Pihlanto-Leppäla A., Rantamäki P., Tupasela T. (1998). Impact of processing on bioactive proteins and peptides. Trends Food Sci. Technol..

[22] Frøkjaer S. (1994). Use of hydrosylates for protein supplementation. Food Technol..

[23] Nakai S., Modler H.W. (1996). Food Proteins: Properties and Characterization.

[24] Koyande A.K., Chew K.W., Rambabu K., Tao Y., Chu D.-T., Show P.-L. (2019). Microalgae: A potential alternative to health supplementation for humans. Food Sci. Hum. Wellness.

[25] Sathivel S., Bechtel P.J. (2006). Properties of soluble protein powders from Alaska pollock (*Theragra chalcogramma*). Int. J. Food Sci. Technol..

[26] Niki H., Deya E., Kato T., Igarashi S. (1982). The process of producing active fish protein powder. Bull. Jap. Soc. Sci. Fish..

[27] Yoon I.S., Lee H.J., Kang S.I., Park S.Y., Kang Y.M., Kim J.S., Heu M.S. (2019). Food functionality of protein isolates extracted from Yellowfin tuna (*Thunnus albacares*) roe using alkaline solubilization and acid precipitation process. Food Sci. Nutr..

[28] Kadam S.U., Prabhasankar P. (2010). Marine foods as functional ingredients in bakery pasta products. Food Res. Int..

[29] Vijaykrishnaraj M., Roopa B., Prabhasankar P. (2016). Preparation of gluten free bread enriched with green mussel (*Perna canaliculus*) protein hydrolysates and characterization of peptides responsible for mussel flavour. Food Chem..

[30] Tacon A.G., Metian M. (2013). Fish matters: Importance of aquatic foods in human nutrition and global food supply. Rev. Fish. Sci..

[31] Wong C.P., Bray T.M., Khanna S.K. (2019). Growth, bone health, and cognition: Nutritional evaluation of a sustainable ocean-based advance protein powder (APP). Ecol. Food Nutr..

[32] Kristinsson H.G., Rasco B.A. (2000). Fish protein hydrolysates: Production, biochemical and functional properties. Crit. Rev. Food Sci. Nutr..

[33] Kent G. (2019). Fish, Food, and Hunger: The Potential of Fisheries for Alleviating Malnutrition.

[34] Da Silva C.P., Bezerra R.S., dos Santos A.C.O., Messias J.B., de Castro C.R.O.B., Junior L.B.C. (2017). Biological value of shrimp protein hydrolysate by-product produced by autolysis. LWT Food Sci. Technol..

[35] Pires C., Batista I., Fradinho P., Costa S. (2009). Utilization of alkaline-recovered proteins from cape hake by-products in the preparation of Frankfurter-type fish sausages. J. Aquat. Food Prod. Technol..

[36] Harrison R.W., Stringer T., Prinyawiwatkul W. (2001). Evaluating consumer preferences for aquacultural products: An application to the US crawfish industry. Aquac. Econ. Manag..

[37] Taskaya L., Jaczynski J. (2009). Flocculation-enhanced protein recovery from fish processing by-products by isoelectric solubilization/precipitation. LWT-Food Sci. Technol..

[38] Tahergorabi R., Beamer S.K., Matak K.E., Jaczynski J. (2012). Functional food products made from fish protein isolate recovered with isoelectric solubilization/precipitation. Food Sci. Technol..

[39] Wijaya W., Patel A.R., Setiowati A.D., Van der Meeren P. (2017). Functional colloids from proteins and polysaccharides for food applications. Trends Food Sci. Technol..

[40] Hu X., Cebe P., Weiss A.S., Omenetto F., Kaplan D.L. (2012). Protein-based composite materials. Mater. Today.

[41] Kim S.K., Mendis E. (2006). Bioactive compounds from marine processing byproducts–A review. Food Res. Int..

[42] Je J.-Y., Park P.-J., Kwon J.Y., Kim S.-K. (2004). A novel angiotensin I converting enzyme inhibitory peptide from Alaska pollack (*Theragra chalcogramma*) frame protein hydrolysate. J. Agric. Food Chem..

[43] Je J.-Y., Kim S.-Y., Kim S.-K. (2005). Preparation and antioxidative activity of hoki frame protein hydrolysate using ultrafiltration membranes. Eur. Food Res. Technol..

[44] Huang S.-L., Jao C.-L., Ho K.-P., Hsu K.-C., Martinez J., Fehrentz J.-A. (2012). Dipeptidyl-Peptidase IV Inhibitory Activity of Peptides Derived from Tuna Cooking Juice Hydrolysates, Peptides 2000. Proceedings of the European Peptide Symposium 26th, Montpellier, France, 10–15 September 2000.

[45] Vilas Boas L.C.P., de Lima L.M.P., Migliolo L., Mendes G.d.S., de Jesus M.G., Franco O.L., Silva P.A. (2017). Linear antimicrobial peptides with activity against herpes simplex virus 1 and Aichi virus. Pept. Sci..

[46] Abdou A.M., Higashiguchi S., Horie K., Kim M., Hatta H., Yokogoshi H. (2006). Relaxation and immunity enhancement effects of γ-Aminobutyric acid (GABA) administration in humans. Biofactors.

[47] Fugelli K. (1970). Gamma-aminobutyric acid (GABA) in fish erythrocytes. Experientia.

[48] Nakajima K., Yoshie-Stark Y., Ogushi M. (2009). Comparison of ACE inhibitory and DPPH radical scavenging activities of fish muscle hydrolysates. Food Chem..

[49] Shao A., Hathcock J.N. (2008). Risk assessment for the amino acids taurine, L-glutamine and L-arginine. Regul. Toxicol. Pharmacol..

[50] Ruiz J., Olivieri G., de Vree J., Bosma R., Willems P., Reith J.H., Eppink M.H., Kleinegris D.M., Wijffels R.H., Barbosa M.J. (2016). Towards industrial products from microalgae. Energy Environ. Sci..

[51] Cavonius L.R., Albers E., Undeland I. (2015). pH-shift processing of *Nannochloropsis oculata* microalgal biomass to obtain a protein-enriched food or feed ingredient. Algal Res..

[52] Gençdağ E., Görgüç A., Yılmaz F.M. (2020). Recent advances in the recovery techniques of plant-based proteins from agro-industrial by-products. Food Rev. Int..

[53] Olsen R.L., Toppe J., Karunasagar I. (2014). Challenges and realistic opportunities in the use of by-products from processing of fish and shellfish. Trends Food Sci. Technol..

[54] Vermuë M., Eppink M., Wijffels R., Van Den Berg C. (2018). Multi-product microalgae biorefineries: From concept towards reality. Trends Biotechnol..

[55] Nguyen T.T. (2017). Biorefinery Process Development for Recovery of Functional and Bioactive Compounds from Lobster Processing by-Products for Food and Nutraceutical Applications.

[56] Yan N., Chen X. (2015). Don’t waste seafood waste. Nature.

[57] Denise S., Jason B. (2012). Food Grade Astaxanthin from Lobster Shell Discards.

[58] Arason S., Karlsdottir M., Valsdottir T., Slizyte R., Rustad T., Falch E., Eysturskard J., Jakobsen G. (2010). Technical Report: Maximum Resource Utilisation-Value Added Fish by-Products.

[59] Fletcher R. (2018). Don’t Bypass the Value of Aquaculture by-Products. Fish. Site.

[60] Nguyen T.T., Barber R.A., Corbin K., Zhang W. (2017). Lobster processing by-products as valuable bioresource of marine functional ingredients, nutraceuticals, and pharmaceuticals. Bioresour. Bioprocess..

[61] Šilovs M. (2018). Fish processing by-products exploitation and innovative fish-based food production. Res. Rural Dev..

[62] Hamed I., Özogul F., Regenstein J.M. (2016). Industrial applications of crustacean by-products (chitin, chitosan, and chitooligosaccharides): A review. Trends Food Sci. Technol..

[63] Chandrasekaran M. (2013). Valorization of Food Processing by-Products.

[64] Suresh P., Kudre T.G., Johny L.C., Singhania R.R., Agarwal R.A., Kumar R.P., Sukumaran R.K. (2018). Sustainable valorization of seafood processing by-product/discard. Waste to Wealth.

[65] Pateiro M., Munekata P.E., Domínguez R., Wang M., Barba F.J., Bermúdez R., Lorenzo J.M. (2020). Nutritional profiling and the value of processing by-products from gilthead sea bream (*Sparus aurata*). Mar. Drugs.

[66] Wu T.H., Nigg J.D., Stine J.J., Bechtel P.J. (2011). Nutritional and chemical composition of by-product fractions produced from wet reduction of individual red salmon (*Oncorhynchus nerka*) heads and viscera. J. Aquat. Food Prod. Technol..

[67] Shruthy R., Preetha R., Goyal M., Suleria H., Kirubanandan S. (2019). Utilization of fish and shellfish byproducts from Marine food Industries: Benefits and challenges. Technological Processes for Marine Foods, from Water to Fork: Bioactive Compounds, Industrial Applications, Genomics.

[68] Blanco M., Vázquez J.A., Pérez-Martín R.I., Sotelo C.G. (2017). Hydrolysates of fish skin collagen: An opportunity for valorizing fish industry byproducts. Mar. Drugs.

[69] Martínez-Alvarez O., Chamorro S., Brenes A. (2015). Protein hydrolysates from animal processing by-products as a source of bioactive molecules with interest in animal feeding: A review. Food Res. Int..

[70] Toppe J., Albrektsen S., Hope B., Aksnes A. (2007). Chemical composition, mineral content and amino acid and lipid profiles in bones from various fish species. Comp. Biochem. Physiol. Part. B Biochem. Mol. Biol..

[71] Masood Z., Yasmeen R., Haider M.S., Tarar O.M., Hossain M. (2015). Evaluations of crude protein and amino acid contents from the scales of four mullet species (*Mugilidae*) collected from Karachi fish harbour, Pakistan. Ind. J. Geo-Mar. Sci..

[72] Cardoso C., Nunes M.L., Galvez R., Berge J.-P. (2013). Improved utilization of fish waste, discards, and by-products and low-value fish towards food and health products. Utilization of Fish Waste.

[73] Hayes M., Gallagher M. (2019). Processing and recovery of valuable components from pelagic blood-water waste streams: A review and recommendations. J. Clean. Prod..

[74] Chernyavskikh S.D., Borodaeva Z.A., Borisovskiy I.P., Ostapenko S.I., Galtseva O.A. (2019). Blood protein spectrum in representatives of the fish superclass. Eurasianj. Biosci..

[75] Rødde R.H., Einbu A., Vårum K.M. (2008). A seasonal study of the chemical composition and chitin quality of shrimp shells obtained from northern shrimp (*Pandalus borealis*). Carbohydr. Polym..

[76] Hossain M., Shikha F., Sharma A. (2018). Waste management status of shrimp processing plants of south and south-west region of Bangladesh. J. Environ. Sci. Nat. Resour..

[77] Trung T.S., Phuong P.T.D. (2012). Bioactive compounds from by-products of shrimp processing industry in Vietnam. J. Food Drug Anal..

[78] Nguyen T.T., Zhang W., Barber R.A., Su P., He S. (2015). Significant enrichment of polyunsaturated fatty acids (PUFAs) in the lipids extracted by supercritical CO_2_ from the livers of Australian rock lobsters *(Jasus edwardsii)*. J. Agric. Food Chem..

[79] Morris J.P., Backeljau T., Chapelle G. (2019). Shells from aquaculture: A valuable biomaterial, not a nuisance waste product. Rev. Aquac..

[80] Marin F., Luquet G. (2004). Molluscan shell proteins. Comptes Rendus Palevol.

[81] Huang G., Bi X., Zhang B., Qu T., Liu B., Fan S., Yu D. (2017). Expression, purification, and functional activity of shell matrix protein pearlin from the pearl oyster *Pinctada fucata*. J. Shellfish Res..

[82] Naik A., Hayes M. (2019). Bioprocessing of mussel by-products for value added ingredients. Trends Food Sci. Technol..

[83] Kumar V., Muzaddadi A.U., Mann S., Balakrishnan R., Bembem K., Kalnar Y. (2018). Utilization of fish processing waste: A waste to wealth approach. Emerg. Post-Harvest Eng. Techological Interv. Enhancing Farmer’s Income.

[84] Derby C.D. (2014). Cephalopod ink: Production, chemistry, functions and applications. Mar. Drugs.

[85] Jose J., Krishnakumar K., Dineshkumar B. (2018). Squid ink and its pharmacological activities. GSC Biol. Pharm. Sci..

[86] Sugiyama M., Hanabe M. (1989). Utilization of Squid.

[87] Singh A., Mittal A., Benjakul S. (2020). Full utilization of squid meat and its processing by-products: Revisit. Food Rev. Int..

[88] Amarowicz R., Synowiecki J., Shahidi F. (2012). Chemical composition of shells from red (*Strongylocentrotus franciscanus*) and green (*Strongylocentrotus droebachiensis*) sea urchin. Food Chem..

[89] Hossain A., Dave D., Shahidi F. (2020). Northern sea cucumber (*Cucumaria frondosa*): A potential candidate for functional food, nutraceutical, and pharmaceutical sector. Mar. Drugs.

[90] Brotz L., Schiariti A., López-Martínez J., Álvarez-Tello J., Hsieh Y.-H.P., Jones R.P., Quiñones J., Dong Z., Morandini A.C., Preciado M. (2017). Jellyfish fisheries in the Americas: Origin, state of the art, and perspectives on new fishing grounds. Rev. Fish. Biol. Fish..

[91] Liu C., Li S., Kong J., Liu Y., Wang T., Xie L., Zhang R. (2015). In-depth proteomic analysis of shell matrix proteins of *Pinctada fucata*. Sci. Rep..

[92] Vieira G.H., Martin A.M., Saker-Sampaiao S., Omar S., Goncalves R.C. (1995). Studies on the enzymatic hydrolysis of Brazilian lobster (*Panulirus* spp.) processing wastes. J. Sci. Food Agric..

[93] Bechtel P.J., Watson M.A., Lea J.M., Bett-Garber K.L., Bland J.M. (2019). Properties of bone from catfish heads and frames. Food Sci. Nutr..

[94] Mace O.J., Morgan E.L., Affleck J.A., Lister N., Kellett G.L. (2007). Calcium absorption by Cav1. 3 induces terminal web myosin II phosphorylation and apical GLUT2 insertion in rat intestine. J. Physiol..

[95] Muralidharan N., Shakila R.J., Sukumar D., Jeyasekaran G. (2013). Skin, bone and muscle collagen extraction from the trash fish, leather jacket (*Odonus niger*) and their characterization. J. Food Sci. Technol..

[96] Xu S., Yang H., Shen L., Li G. (2017). Purity and yield of collagen extracted from southern catfish (*Silurus meridionalis* Chen) skin through improved pretreatment methods. Int. J. Food Prop..

[97] Benjakul S., Oungbho K., Visessanguan W., Thiansilakul Y., Roytrakul S. (2009). Characteristics of gelatin from the skins of bigeye snapper, *Priacanthus tayenus* and *Priacanthus macracanthus*. Food Chem..

[98] Binsi P., Shamasundar B., Dileep A., Badii F., Howell N. (2009). Rheological and functional properties of gelatin from the skin of Bigeye snapper (*Priacanthus hamrur*) fish: Influence of gelatin on the gel-forming ability of fish mince. Food Hydrocoll..

[99] Sukkwai S., Kijroongrojana K., Benjakul S. (2011). Extraction of gelatin from bigeye snapper (*Priacanthus tayenus*) skin for gelatin hydrolysate production. Int. Food Res. J..

[100] Aewsiri T., Benjakul S., Visessanguan W. (2009). Functional properties of gelatin from cuttlefish (*Sepia pharaonis*) skin as affected by bleaching using hydrogen peroxide. Food Chem..

[101] Nagai T., Araki Y., Suzuki N. (2002). Collagen of the skin of ocellate puffer fish (*Takifugu rubripes*). Food Chem..

[102] Kołodziejska I., Sikorski Z.E., Niecikowska C. (1999). Parameters affecting the isolation of collagen from squid (*Illex argentinus*) skins. Food Chem..

[103] Senaratne L., Park P.-J., Kim S.-K. (2006). Isolation and characterization of collagen from brown backed toadfish (*Lagocephalus gloveri*) skin. Bioresour. Technol..

[104] Nagai T., Suzuki N. (2000). Isolation of collagen from fish waste material—Skin, bone and fins. Food Chem..

[105] Kittiphattanabawon P., Benjakul S., Visessanguan W., Nagai T., Tanaka M. (2005). Characterisation of acid-soluble collagen from skin and bone of bigeye snapper (*Priacanthus tayenus*). Food Chem..

[106] Aewsiri T., Benjakul S., Visessanguan W., Tanaka M. (2008). Chemical compositions and functional properties of gelatin from pre-cooked tuna fin. Int. J. Food Sci..

[107] Okazaki E., Osako K. (2014). Isolation and characterization of acid-soluble collagen from the scales of marine fishes from Japan and Vietnam. Food Chem..

[108] Villamil O., Váquiro H., Solanilla J.F. (2017). Fish viscera protein hydrolysates: Production, potential applications and functional and bioactive properties. Food Chem..

[109] Ezquerra-Brauer J.M., Haard N.F., Ramirez-Olivas R., Olivas-Burrola H., Vela zquez-Sanchez C.J. (2002). Influence of harvest season on the proteolytic activity of hepatopancreas and mantle tissues from jumbo squid (*Doswicus gigas*). J. Food Biochem..

[110] FAO (2018). The State of World Fisheries and Aquaculture 2018-Meeting the Sustainable Development Goals.

[111] Kumar N.S., Nazeer R., Jaiganesh R. (2011). Purification and Biochemical Characterization of Antioxidant Peptide from Horse Mackerel (Magalaspis Cordyla) Viscera Protein, Peptides 2000. Proceedings of the European Peptide Symposium 26th, Montpellier, France, 10–15 September 2000.

[112] Sriket C. (2014). Proteases in fish and shellfish: Role on muscle softening and prevention. Int. Food Res. J..

[113] Shahidi F., Kamil Y.J. (2001). Enzymes from fish and aquatic invertebrates and their application in the food industry. Trends Food Sci. Technol..

[114] Raa J. (1990). Biotechnology in Aquaculture and the Fish Processing Industry: A Success Story in Norway.

[115] North M., Beynon R.J., Bond J.S. (1989). Prevention of unwanted proteolysis. Proteolytic Enzymes: A Practical Approach.

[116] Bezerra R.S., Lins E.J., Alencar R.B., Paiva P.M., Chaves M.E., Coelho L.C., Carvalho L.B. (2005). Alkaline proteinase from intestine of Nile tilapia (*Oreochromis niloticus*). Process Biochem..

[117] Klomklao S., Kishimura H., Nonami Y., Benjakul S. (2009). Biochemical properties of two isoforms of trypsin purified from the intestine of skipjack tuna (*Katsuwonus pelamis*). Food Chem..

[118] Gildberg A. (1992). Recovery of proteinases and protein hydrolysates from fish viscera. Bioresour. Technol..

[119] Murthy L.N., Phadke G.G., Unnikrishnan P., Annamalai J., Joshy C.G., Zynudheen A.A., Ravishankar C.N. (2018). Valorization of fish viscera for crude proteases production and its use in bioactive protein hydrolysate preparation. Waste Biomass Valoriz..

[120] Ketnawa S., Benjakul S., Ling T.C., Martínez-Alvarez O., Rawdkuen S. (2013). Enhanced recovery of alkaline protease from fish viscera by phase partitioning and its application. Chem. Cent. J..

[121] Bezerra R., Santos J.F., Paiva P.M., Correia M.T., Coelho L.C., Vieira V.L., Carvalho JR L.B. (2001). Partial purification and characterization of a thermostable trypsin from pyloric caeca of tambaqui (*Colossoma macropomum*). J. Food Biochem..

[122] Khantaphant S., Benjakul S. (2010). Purification and characterization of trypsin from the pyloric caeca of brownstripe red snapper (*Lutjanus vitta*). Food Chem..

[123] Klomklao S., Benjakul S., Visessanguan W., Kishimura H., Simpson B.K. (2007). Trypsin from the pyloric caeca of bluefish (*Pomatomus saltatrix*). Comp. Biochem. Physiol. Part B Biochem. Mol. Biol..

[124] Silva J.F., Espósito T.S., Marcuschi M., Ribeiro K., Cavalli R.O., Oliveira V., Bezerra R.S. (2011). Purification and partial characterisation of a trypsin from the processing waste of the silver mojarra (*Diapterus rhombeus*). Food Chem..

[125] Travis J., Salvesen G. (1983). Human plasma proteinase inhibitors. Ann. Rev. Biochem..

[126] Li D., Lin H., Kim S. (2008). Effect of rainbow trout (*Oncorhynchus mykiss*) plasma protein on the gelation of Alaska pollock (*Theragra chalcogramma*) surimi. J. Food Sci..

[127] Li D.K., Lin H., Kim S.M. (2008). Purification and characterization of a cysteine protease inhibitor from chum salmon (*Oncorhynchus keta*) plasma. J. Agric. Food Chem..

[128] Fowler M.R., Park J.W. (2015). Salmon blood plasma: Effective inhibitor of protease-laden Pacific whiting surimi and salmon mince. Food Chem..

[129] Barrett A.J. (1981). [54] α2-Macroglobulin. Methods in Enzymology.

[130] Simke A. (2020). Fish Blood May Provide Biotech Researchers with an Ethical Alternative to Fetal Bovine Fluid. Forbes.

[131] Tadashi M., Haruko T., Miyashita H., Hiroko Y. (2005). Marine microalgae. Adv. Biochem. Engin. Biotechnol..

[132] Vigani M., Parisi C., Rodríguez-Cerezo E., Barbosa M.J., Sijtsma L., Ploeg M., Enzing C. (2015). Food and feed products from micro-algae: Market opportunities and challenges for the EU. Trends Food Sci. Technol..

[133] William M., Asaf T. (2017). Micro Solutions for a Macro Problem: How Marine Algae Could Help Feed the World. Conversation.

[134] Bleakley S., Hayes M. (2017). Algal proteins: Extraction, application, and challenges concerning production. Foods.

[135] Velu C., Cirés S., Brinkman D.L., Heimann K. (2020). Bioproduct potential of outdoor cultures of *Tolypothrix* sp.: Effect of carbon dioxide and metal-rich wastewater. Front. Bioeng. Biotechnol..

[136] Christaki E., Florou-Paneri P., Bonos E. (2011). Microalgae: A novel ingredient in nutrition. Int. J. Food Sci. Nutr..

[137] Richmond A. (2008). Handbook of Microalgal Culture: Biotechnology and Applied Phycology.

[138] Van Krimpen M., Bikker P., Van der Meer I., Van der Peet-Schwering C., Vereijken J. (2013). Cultivation, Processing and Nutritional Aspects for Pigs and Poultry of European Protein Sources as Alternatives for Imported Soybean Products.

[139] Hur S.B., Bae J.H., Youn J.-Y., Jo M.J. (2015). KMMCC-Korea marine microalgae culture center: List of strains. Algae.

[140] Mogharabi M., Faramarzi M.A. (2016). Are algae the future source of enzymes?. Trends Pept. Prot. Sci..

[141] Brasil B.D.S.A.F., de Siqueira F.G., Salum T.F.C., Zanette C.M., Spier M.R. (2017). Microalgae and cyanobacteria as enzyme biofactories. Algal Res..

[142] Gong Y., Hu H., Gao Y., Xu X., Gao H. (2011). Microalgae as platforms for production of recombinant proteins and valuable compounds: Progress and prospects. J. Ind. Microbiol. Biotechnol..

[143] Fox R.D. (1996). Spirulina: Production & Potential.

[144] Belay A. (2002). The potential application of *Spirulina* (*Arthrospira*) as a nutritional and therapeutic supplement in health management. Am. Nutraceut. Assoc..

[145] Vingiani G.M., De Luca P., Ianora A., Dobson A.D., Lauritano C. (2019). Microalgal enzymes with biotechnological applications. Mar. Drugs.

[146] Becker E. (2007). Microalgae as a source of protein. Biotechnol. Adv..

[147] Tibbetts S.M., Patelakis S.J., Whitney-Lalonde C.G., Garrison L.L., Wall C.L., MacQuarrie S.P. (2019). Nutrient composition and protein quality of microalgae meals produced from the marine prymnesiophyte *Pavlova* sp. 459 mass-cultivated in enclosed photobioreactors for potential use in salmonid aquafeeds. J. Appl. Phycol..

[148] Tahergorabi R., Jaczynski J., Raatz S., Bibus D. (2016). Seafood proteins and human health. Fish and Fish Oil in Health and Disease Prevention.

[149] Peng S., Chen C., Shi Z., Wang L. (2013). Amino acid and fatty acid composition of the muscle tissue of yellowfin tuna (*Thunnus albacares*) and bigeye tuna (*Thunnus obesus*). J. Food Nutr. Res..

[150] Leal A.L.G., de Castro P.F., de Lima J.P.V., de Souza Correia E., de Souza Bezerra R. (2010). Use of shrimp protein hydrolysate in Nile tilapia (*Oreochromis niloticus*, L.) feeds. Aquac. Int..

[151] Barka A., Blecker C. (2016). Microalgae as a potential source of single-cell proteins. A review. Biotechnol. Agron. Soc. Environ..

[152] Kent M., Welladsen H.M., Mangott A., Li Y. (2015). Nutritional evaluation of Australian microalgae as potential human health supplements. PLoS ONE.

[153] Lim A.S., Jeong H.J., Kim S.J., Ok J.H., Lim A.S., Jeong H.J., Kim S.J., Ok J.H. (2018). Amino acids profiles of six dinoflagellate species belonging to diverse families: Possible use as animal feeds in aquaculture. Algae.

[154] Cao W., Zhang C., Hong P., Ji H. (2008). Response surface methodology for autolysis parameters optimization of shrimp head and amino acids released during autolysis. Food Chem..

[155] Niittynen L., Nurminen M.-L., Korpela R., Vapaatalo H. (1999). Role of arginine, taurine 4 and homocysteine in cardiovascular diseases. Ann. Med..

[156] Oomah B., Mazza G., Francis F. (2000). Functional foods. The Wiley Encyclopedia of Science and Technology.

[157] Sidransky H. (1990). Possible role of dietary proteins and amino acids in atherosclerosis. Ann. N. Y. Acad. Sci..

[158] Beauchamp G.K. (2009). Sensory and receptor responses to umami: An overview of pioneering work. Am. J. Clin. Nutr..

[159] Yamaguchi S. (1998). Basic properties of umami and its effects on food flavor. Food Rev. Int..

[160] Konasu S., Yamaguchi K., Hebard C., Flick G., Martin R. (1982). Trimethylamine contents in fishery products. Chemistry and Biochemistry of Marine Food Products.

[161] Guichard E., Salles C., Elisabeth G., Christan S., Andree V., Patrick E. (2016). Retention and release of taste and aroma compounds from the food matrix during mastication and ingestion. Flavor: From Food to Behaviors, Wellbeing and Health.

[162] Imm J., Lee C. (1999). Production of seafood flavor from red hake (*Urophycis chuss*) by enzymatic hydrolysis. J. Agric. Food Chem..

[163] Korhonen H., Pihlanto A. (2006). Bioactive peptides: Production and functionality. Int. Dairy J..

[164] Udenigwe C.C., Aluko R.E. (2012). Food protein-derived bioactive peptides: Production, processing, and potential health benefits. J. Food Sci..

[165] Bouglé D., Bouhallab S. (2017). Dietary bioactive peptides: Human studies. Crit. Rev. Food Sci. Nutr..

[166] Jo C., Khan F.F., Khan M.I., Iqbal J. (2017). Marine bioactive peptides: Types, structures, and physiological functions. Food Rev. Int..

[167] Ishak N., Sarbon N. (2018). A review of protein hydrolysates and bioactive peptides deriving from wastes generated by fish processing. Food Bioprocess. Technol..

[168] Lee S.-H., Qian Z.-J., Kim S.-K. (2010). A novel angiotensin I converting enzyme inhibitory peptide from tuna frame protein hydrolysate and its antihypertensive effect in spontaneously hypertensive rats. Food Chem..

[169] Du L., Fang M., Wu H., Xie J., Wu Y., Li P., Zhang D., Huang Z., Xia Y., Zhou L. (2013). A novel angiotensin I-converting enzyme inhibitory peptide from *Phascolosoma esculenta* water-soluble protein hydrolysate. J. Function. Foods.

[170] Qian Z.-J., Heo S.-J., Oh C.H., Kang D.-H., Jeong S.H., Park W.S., Choi I.-W., Jeon Y.-J., Jung W.-K. (2013). Angiotensin I-converting enzyme (ACE) inhibitory peptide isolated from biodiesel byproducts of marine microalgae, *Nannochloropsis oculata*. J. Biobased Mater. Bioenergy.

[171] He H.-L., Chen X.-L., Wu H., Sun C.-Y., Zhang Y.-Z., Zhou B.-C. (2007). High throughput and rapid screening of marine protein hydrolysates enriched in peptides with angiotensin-I-converting enzyme inhibitory activity by capillary electrophoresis. Bioresour. Technol..

[172] Pujiastuti D.Y., Amin G., Nur M., Alamsjah M.A., Hsu J.-L. (2019). Marine organisms as potential sources of bioactive peptides that inhibit the activity of angiotensin I-converting enzyme: A review. Molecules.

[173] Wu J., Aluko R.E., Nakai S. (2006). Structural requirements of angiotensin I-converting enzyme inhibitory peptides: Quantitative structure− activity relationship study of di-and tripeptides. J. Agric. Food Chem..

[174] Wang Q., Wang Q. (2018). Preparation of functional peanut oligopeptide and its biological activity. Peanut Processing Characteristics and Quality Evaluation.

[175] Zhou D.Y., Liu Z.Y., Zhao J., Xi M.Z., Fu Y.H., Zhang T., Ji C.F., Zhu B.W. (2017). Antarctic krill (*Euphausia superba*) protein hydrolysates stimulate cholecystokinin release in STC-1 cells and its signaling mechanism. J. Food Process. Preserv..

[176] He H.-L., Liu D., Ma C.-B. (2013). Review on the angiotensin-I-converting enzyme (ACE) inhibitor peptides from marine proteins. Appl. Biochem. Biotechnol..

[177] Samarakoon K.W., Kwon O.-N., Ko J.-Y., Lee J.-H., Kang M.-C., Kim D., Lee J.B., Lee J.-S., Jeon Y.-J. (2013). Purification and identification of novel angiotensin-I converting enzyme (ACE) inhibitory peptides from cultured marine microalgae (*Nannochloropsis oculata*) protein hydrolysate. J. Appl. Phycol..

[178] Samarakoon K., Ko S.-C., You-Jin J. Isolation and purification of angiotensin-I converting enzyme (ACE) inhibitory peptides from marine microalgae. Proceedings of the International Conference on Fisheries & Marine Science (Marine Fish 2012).

[179] Wu H., Xu N., Sun X., Yu H., Zhou C. (2015). Hydrolysis and purification of ACE inhibitory peptides from the marine microalgae *Isochrysis galbana*. J. Appl. Phycol..

[180] Sun S., Xu X., Sun X., Zhang X., Chen X., Xu N. (2019). Preparation and identification of ACE inhibitory peptides from the marine macroalga *Ulva intestinalis*. Mar. Drugs.

[181] Saito T., Bösze Z. (2008). Antihypertensive peptides derived from bovine casein and whey proteins. Bioactive Components of Milk.

[182] Nakashima Y., Arihara K., Sasaki A., Mio H., Ishikawa S., Itoh M. (2002). Antihypertensive activities of peptides derived from porcine skeletal muscle myosin in spontaneously hypertensive rats. J. Food Sci..

[183] Stadnik J., Kęska P. (2015). Meat and fermented meat products as a source of bioactive peptides. Acta Sci. Polon. Technol. Aliment..

[184] Davalos A., Miguel M., Bartolome B., Lopez-Fandino R. (2004). Antioxidant activity of peptides derived from egg white proteins by enzymatic hydrolysis. J. Food Protect..

[185] Singh B.P., Vij S., Hati S. (2014). Functional significance of bioactive peptides derived from soybean. Peptides.

[186] Park S.Y., Je J.-Y., Ahn C.-B. (2016). Protein hydrolysates and ultrafiltration fractions obtained from krill (*Euphausia superba*): Nutritional, functional, antioxidant, and ACE-Inhibitory characterization. J. Aquat. Food Prod. Technol..

[187] Hai-Lun H., Xiu-Lan C., Cai-Yun S., Yu-Zhong Z., Bai-Cheng Z. (2006). Analysis of novel angiotensin-I-converting enzyme inhibitory peptides from protease-hydrolyzed marine shrimp *Acetes Chinensis*. J. Pept. Sci..

[188] Chizuru S., Satoshi T., Riho T., Saki F., Miyu K., Chikako A., Yoshitoshi N. (2019). Isolation and identification of an angiotensin I-converting enzyme inhibitory peptide from pearl oyster (*Pinctada fucata*) shell protein hydrolysate. Process Biochem..

[189] Noorani K.P.M., Nazeer R. (2020). Enzymatic production of two tri-peptides on ACE-I Inhibition and antioxidant activities. Int. J. Pept. Res. Therapeut..

[190] Liu X., Zhang M., Shi Y., Qiao R., Tang W., Sun Z. (2016). Production of the angiotensin I converting enzyme inhibitory peptides and isolation of four novel peptides from jellyfish (*Rhopilema esculentum*) protein hydrolysate. J. Sci. Food Agric..

[191] Zhao Y., Li B., Dong S., Liu Z., Zhao X., Wang J., Zeng M. (2009). A novel ACE Inhibitory Peptide Isolated from Acaudina Molpadioidea Hydrolysate, Peptides 2000. Proceedings of the European Peptide Symposium 26th, Montpellier, France, 10–15 September 2000.

[192] Balti R., Nedjar-Arroume N., Bougatef A., Guillochon D., Nasri M. (2010). Three novel angiotensin I-converting enzyme (ACE) inhibitory peptides from cuttlefish (*Sepia officinalis*) using digestive proteases. Food Res. Int..

[193] Alemán A., Gómez-Guillén M., Montero P. (2013). Identification of ACE-inhibitory peptides from squid skin collagen after in vitro gastrointestinal digestion. Food Res. Int..

[194] Himaya S., Ngo D.-H., Ryu B., Kim S.-K. (2012). An active peptide purified from gastrointestinal enzyme hydrolysate of Pacific cod skin gelatin attenuates angiotensin-1 converting enzyme (ACE) activity and cellular oxidative stress. Food Chem..

[195] Neves A.C., Harnedy P.A., O’Keeffe M.B., Alashi M.A., Aluko R.E., FitzGerald R.J. (2017). Peptide identification in a salmon gelatin hydrolysate with antihypertensive, dipeptidyl peptidase IV inhibitory and antioxidant activities. Food Res. Int..

[196] Hayes M., Mora L., Hussey K., Aluko R.E. (2016). Boarfish protein recovery using the pH-shift process and generation of protein hydrolysates with ACE-I and antihypertensive bioactivities in spontaneously hypertensive rats. Innov. Food Sci. Emerg. Technol..

[197] Salampessy J., Reddy N., Phillips M., Kailasapathy K. (2017). Isolation and characterization of nutraceutically potential ACE-Inhibitory peptides from leatherjacket (*Meuchenia* sp.) protein hydrolysates. LWT-Food Sci. Technol..

[198] Ko J.-Y., Kang N., Lee J.-H., Kim J.-S., Kim W.-S., Park S.-J., Kim Y.-T., Jeon Y.-J. (2016). Angiotensin I-converting enzyme inhibitory peptides from an enzymatic hydrolysate of flounder fish (*Paralichthys olivaceus*) muscle as a potent anti-hypertensive agent. Process Biochem..

[199] Kim H.-J., Park K.-H., Shin J.-H., Lee J.-S., Heu M.-S., Lee D.-H., Kim J.-S. (2011). Antioxidant and ACE inhibiting activities of the rockfish *Sebastes hubbsi* skin gelatin hydrolysates produced by sequential two-step enzymatic hydrolysis. Fish. Aquat. Sci..

[200] Ngo D.-H., Ryu B., Kim S.-K. (2014). Active peptides from skate (*Okamejei kenojei*) skin gelatin diminish angiotensin-I converting enzyme activity and intracellular free radical-mediated oxidation. Food Chem..

[201] Je J.-Y., Park J.-Y., Jung W.-K., Park P.-J., Kim S.-K. (2005). Isolation of angiotensin I converting enzyme (ACE) inhibitor from fermented oyster sauce, *Crassostrea gigas*. Food Chem..

[202] Alemán A., Pérez-Santín E., Bordenave-Juchereau S., Arnaudin I., Gómez-Guillén M., Montero P. (2011). Squid gelatin hydrolysates with antihypertensive, anticancer and antioxidant activity. Food Res. Int..

[203] Ko S.-C., Kang N., Kim E.-A., Kang M.C., Lee S.-H., Kang S.-M., Lee J.-B., Jeon B.-T., Kim S.-K., Park S.-J. (2012). A novel angiotensin I-converting enzyme (ACE) inhibitory peptide from a marine Chlorella ellipsoidea and its antihypertensive effect in spontaneously hypertensive rats. Process Biochem..

[204] Lin Y.-H., Chen G.-W., Yeh C.H., Song H., Tsai J.-S. (2018). Purification and identification of angiotensin I-converting enzyme inhibitory peptides and the antihypertensive effect of *Chlorella sorokiniana* protein hydrolysates. Nutrients.

[205] Montone C.M., Capriotti A.L., Cavaliere C., La Barbera G., Piovesana S., Chiozzi R.Z., Laganà A. (2018). Peptidomic strategy for purification and identification of potential ACE-inhibitory and antioxidant peptides in *Tetradesmus obliquus* microalgae. Anal. Bioanal. Chem..

[206] Tejano L.A., Peralta J.P., Yap E.E.S., Chang Y.W. (2019). Bioactivities of enzymatic protein hydrolysates derived from *Chlorella sorokiniana*. Food Sci. Nutr..

[207] Larsen R., Stormo S.K., Dragnes B.T., Elvevoll E.O. (2007). Losses of taurine, creatine, glycine and alanine from cod (*Gadus morhua* L.) fillet during processing. J. Food Comp. Anal..

[208] Lourenco R., Camilo M. (2002). Taurine: A conditionally essential amino acid in humans? An overview in health and disease. Nutr. Hosp..

[209] Militante J., Lombardini J. (2002). Treatment of hypertension with oral taurine: Experimental and clinical studies. Amino Acids.

[210] Zhang M., Bi L., Fang J., Su X., Da G., Kuwamori T., Kagamimori S. (2004). Beneficial effects of taurine on serum lipids in overweight or obese non-diabetic subjects. Amino Acids.

[211] Harnedy P.A., FitzGerald R.J. (2012). Bioactive peptides from marine processing waste and shellfish: A review. J. Funct. Foods.

[212] Owens D.F., Kriegstein A.R. (2002). Is there more to GABA than synaptic inhibition?. Nat. Rev. Neurosci..

[213] Tsai J., Lin Y., Pan B., Chen T. (2006). Antihypertensive peptides and γ-aminobutyric acid from prozyme 6 facilitated lactic acid bacteria fermentation of soymilk. Process Biochem..

[214] Mendis E., Rajapakse N., Byun H.-G., Kim S.-K. (2005). Investigation of jumbo squid (*Dosidicus gigas*) skin gelatin peptides for their in vitro antioxidant effects. Life Sci..

[215] Qian Z.-J., Jung W.-K., Byun H.-G., Kim S.-K. (2008). Protective effect of an antioxidative peptide purified from gastrointestinal digests of oyster, *Crassostrea gigas* against free radical induced DNA damage. Bioresour. Technol..

[216] Rajapakse N., Mendis E., Jung W.-K., Je J.-Y., Kim S.-K. (2005). Purification of a radical scavenging peptide from fermented mussel sauce and its antioxidant properties. Food Res. Int..

[217] Yahia D.A., Madani S., Prost E., Prost J., Bouchenak M., Belleville J. (2003). Tissue antioxidant status differs in spontaneously hypertensive rats fed fish protein or casein. J. Nutr..

[218] Girard A., Prost J.L., Ait-Yahia D., Bouchenak M., Belleville J. (2004). Fish protein improves the total antioxidant status of streptozotocin-induced diabetes in spontaneously hypertensive rat. Med. Sci. Monit..

[219] Jensen I.-J., Walquist M., Liaset B., Elvevoll E.O., Eilertsen K.-E. (2016). Dietary intake of cod and scallop reduces atherosclerotic burden in female apolipoprotein E-deficient mice fed a Western-type high fat diet for 13 weeks. Nutr. Metabol..

[220] Parra D., Bandarra N.M., Kiely M., Thorsdottir I., Martínez J.A. (2007). Impact of fish intake on oxidative stress when included into a moderate energy-restricted program to treat obesity. Eur. J. Nutr..

[221] Seth A., Mahoney R.R. (2001). Iron chelation by digests of insoluble chicken muscle protein: The role of histidine residues. J. Sci. Food Agric..

[222] Kang K.-H., Qian Z.-J., Ryu B., Kim S.-K. (2011). Characterization of growth and protein contents from microalgae *Navicula incerta* with the investigation of antioxidant activity of enzymatic hydrolysates. Food Sci. Biotechnol..

[223] García-Moreno P.J., Batista I., Pires C., Bandarra N.M., Espejo-Carpio F.J., Guadix A., Guadix E.M. (2014). Antioxidant activity of protein hydrolysates obtained from discarded Mediterranean fish species. Food Res. Int..

[224] Mendis E., Rajapakse N., Kim S.-K. (2005). Antioxidant properties of a radical-scavenging peptide purified from enzymatically prepared fish skin gelatin hydrolysate. J. Agric. Food Chem..

[225] Zou T.-B., He T.-P., Li H.-B., Tang H.-W., Xia E.-Q. (2016). The structure-activity relationship of the antioxidant peptides from natural proteins. Molecules.

[226] Nikoo M., Benjakul S., Ehsani A., Li J., Wu F., Yang N., Xu B., Jin Z., Xu X. (2014). Antioxidant and cryoprotective effects of a tetrapeptide isolated from Amur sturgeon skin gelatin. J. Funct. Foods.

[227] Je J.-Y., Qian Z.-J., Byun H.-G., Kim S.-K. (2007). Purification and characterisation of an antioxidant peptide obtained from tuna backbone protein by enzymatic hydrolysis. Process Biochem..

[228] Chi C.-F., Wang B., Hu F.-Y., Wang Y.-M., Zhang B., Deng S.-G., Wu C.-W. (2015). Purification and identification of three novel antioxidant peptides from protein hydrolysate of bluefin leatherjacket (*Navodon septentrionalis*) skin. Food Res. Int..

[229] Song R., Wei R., Zhang B., Yang Z., Wang D. (2011). Antioxidant and antiproliferative activities of heated sterilized pepsin hydrolysate derived from half-fin anchovy (*Setipinna taty*). Mar. Drugs.

[230] Senphan T., Benjakul S. (2014). Antioxidative activities of hydrolysates from seabass skin prepared using protease from hepatopancreas of Pacific white shrimp. J. Funct. Foods.

[231] Karnjanapratum S., Benjakul S., O’callaghan Y., O’Keeffe M., FitzGerald R., O’Brien N. (2016). Purification and identification of antioxidant peptides from gelatin hydrolysates of unicorn leatherjacket skin. Ital. J. Food Sci..

[232] Kim S.M. (2011). Antioxidant and anticancer activities of enzymatic hydrolysates of solitary tunicate (*Styela clava*). Food Sci. Biotechnol..

[233] Kang K.H., Qian Z.J., Ryu B., Karadeniz F., Kim D., Kim S.K. (2012). Antioxidant peptides from protein hydrolysate of microalgae *Navicula incerta* and their protective effects in HepG2/CYP2E1 cells induced by ethanol. Phytother. Res..

[234] Ko S.-C., Kim D., Jeon Y.-J. (2012). Protective effect of a novel antioxidative peptide purified from a marine *Chlorella ellipsoidea* protein against free radical-induced oxidative stress. Food Chem. Toxicol..

[235] Stack J., Gouic A.V.L., Tobin P.R., Guihéneuf F., Stengel D.B., FitzGerald R.J. (2018). Protein extraction and bioactive hydrolysate generation from two microalgae, *Porphyridium purpureum* and *Phaeodactylum tricornutum*. J. Food Bioact..

[236] Ryu B., Kang K.-H., Ngo D.-H., Qian Z.-J., Kim S.-K. (2012). Statistical optimization of microalgae *Pavlova lutheri* cultivation conditions and its fermentation conditions by yeast, *Candida rugopelliculosa*. Bioresour. Technol..

[237] Ouellet V., Weisnagel S.J., Marois J., Bergeron J., Julien P., Gougeon R., Tchernof A., Holub B.J., Jacques H. (2008). Dietary cod protein reduces plasma C-reactive protein in insulin-resistant men and women. J. Nutr..

[238] Zhu C.-F., Li G.-Z., Peng H.-B., Zhang F., Chen Y., Li Y. (2010). Treatment with marine collagen peptides modulates glucose and lipid metabolism in Chinese patients with type 2 diabetes mellitus. Appl. Physiol. Nutr. Metabol..

[239] Kawabata F., Mizushige T., Uozumi K., Hayamizu K., Han L., Tsuji T., Kishida T. (2015). Fish protein intake induces fast-muscle hypertrophy and reduces liver lipids and serum glucose levels in rats. Biosci. Biotechnol. Biochem..

[240] Nasri R., Abdelhedi O., Jemil I., Daoued I., Hamden K., Kallel C., Elfeki A., Lamri-Senhadji M., Boualga A., Nasri M. (2015). Ameliorating effects of goby fish protein hydrolysates on high-fat-high-fructose diet-induced hyperglycemia, oxidative stress and deterioration of kidney function in rats. Chem. Biol. Interact..

[241] Ktari N., Nasri R., Mnafgui K., Hamden K., Belguith O., Boudaouara T., Feki A.E., Nasri M. (2014). Antioxidative and ACE inhibitory activities of protein hydrolysates from zebra blenny (*Salaria basilisca*) in alloxan-induced diabetic rats. Process Biochem..

[242] Khaled H.B., Ghlissi Z., Chtourou Y., Hakim A., Ktari N., Fatma M.A., Barkia A., Sahnoun Z., Nasri M. (2012). Effect of protein hydrolysates from sardinelle (*Sardinella aurita*) on the oxidative status and blood lipid profile of cholesterol-fed rats. Food Res. Int..

[243] Ou Y., Lin L., Pan Q., Yang X., Cheng X.C. (2012). Preventive effect of phycocyanin from *Spirulina platensis* on alloxan-injured mice. Environ. Toxicol. Pharmacol..

[244] Nongonierma A.B., FitzGerald R.J. (2016). Prospects for the management of type 2 diabetes using food protein-derived peptides with dipeptidyl peptidase IV (DPP-IV) inhibitory activity. Curr. Opin. Food Sci..

[245] Xia E.-Q., Zhu S.-S., He M.-J., Luo F., Fu C.-Z., Zou T.-B. (2017). Marine peptides as potential agents for the management of type 2 diabetes mellitus-A prospect. Mar. Drugs.

[246] Li Y., Lammi C., Boschin G., Arnoldi A., Aiello G. (2019). Recent advances in microalgae peptides: Cardiovascular health benefits and analysis. J. Agric. Food Chem..

[247] Beaulieu L., Sirois M., Tamigneaux É. (2016). Evaluation of the in vitro biological activity of protein hydrolysates of the edible red alga, Palmaria palmata (dulse) harvested from the Gaspe coast and cultivated in tanks. J. Appl. Phycol..

[248] Nongonierma A.B., FitzGerald R.J. (2014). An in silico model to predict the potential of dietary proteins as sources of dipeptidyl peptidase IV (DPP-IV) inhibitory peptides. Food Chem..

[249] Zhu C.-F., Peng H.-B., Liu G.-Q., Zhang F., Li Y. (2010). Beneficial effects of oligopeptides from marine salmon skin in a rat model of type 2 diabetes. Nutrition.

[250] Li-Chan E.C., Hunag S.-L., Jao C.-L., Ho K.-P., Hsu K.-C. (2012). Peptides derived from Atlantic salmon skin gelatin as dipeptidyl-peptidase IV inhibitors. J. Agric. Food Chem..

[251] La Rochelle H.D., Courois E., Cudennec B., Fouchereau-Peron M., Ravallec-Ple R. (2011). Fish Protein Hydrolysate Having a Satietogenic Activity, Nutraceutical and Pharmacological Compositions Comprising Such a Hydrolysate and Method for Obtaining Same. U.S. Patent.

[252] Harnedy P.A., Parthsarathy V., McLaughlin C.M., O’Keeffe M.B., Allsopp P.J., McSorley E.M., O’Harte F.P., FitzGerald R.J. (2018). Blue whiting (*Micromesistius poutassou*) muscle protein hydrolysate with in vitro and in vivo antidiabetic properties. J. Funct. Foods.

[253] Roomi M.W., Shanker N., Niedzwiecki A., Rath M. (2015). Induction of apoptosis in the human prostate cancer cell line DU-145 by a novel micronutrient formulation. Open J. Apoptosis.

[254] Guo-Fang D., Huang F.-F., Zui-Su Y., Di Y., Yong-Fang Y. (2011). Anticancer activity of an oligopeptide isolated from hydrolysates of *Sepia* ink. Chin. J. Nat. Med..

[255] Le Gouic A., Harnedy P., FitzGerald R., Mérillon J., Ramawat K. (2018). Bioactive peptides from fish protein by-products. Bioactive Molecules in Food.

[256] Pan X., Zhao Y.-Q., Hu F.-Y., Chi C.-F., Wang B. (2016). Anticancer activity of a hexapeptide from skate (*Raja porosa*) cartilage protein hydrolysate in HeLa Cells. Mar. Drugs.

[257] Chi C.-F., Hu F.-Y., Wang B., Li T., Ding G.-F. (2015). Antioxidant and anticancer peptides from the protein hydrolysate of blood clam (*Tegillarca granosa*) muscle. J. Funct. Foods.

[258] Chalamaiah M., Yu W., Wu J. (2018). Immunomodulatory and anticancer protein hydrolysates (peptides) from food proteins: A review. Food Chem..

[259] Song R., Wei R.-B., Luo H.-Y., Yang Z.-S. (2014). Isolation and identification of an antiproliferative peptide derived from heated products of peptic hydrolysates of half-fin anchovy (*Setipinna taty*). J. Funct. Foods.

[260] Darvish M., Jalili H., Ranaei-Siadat S.-O., Sedighi M. (2018). Potential cytotoxic effects of peptide fractions from *Dunaliella salina* protein hydrolyzed by gastric proteases. J. Aquat. Food Prod. Technol..

[261] Otani H., Suzuki H. (2003). Isolation and characterization of cytotoxic small peptides, α-casecidins, from bovine αs1-casein digested with bovine trypsin. Anim. Sci. J..

[262] Hsu K.-C., Li-Chan E.C.Y., Jao C.-L. (2011). Antiproliferative activity of peptides prepared from enzymatic hydrolysates of tuna dark muscle on human breast cancer cell line MCF-7. Food Chem..

[263] Yang J.-I., Tang J.-Y., Liu Y.-S., Wang H.-R., Lee S.-Y., Yen C.-Y., Chang H.-W. (2016). Roe protein hydrolysates of giant grouper (*Epinephelus lanceolatus*) inhibit cell proliferation of oral cancer cells involving apoptosis and oxidative stress. Biomed. Res. Int..

[264] Picot L., Bordenave S., Didelot S., Fruitier-Arnaudin I., Sannier F., Thorkelsson G., Bergé J., Guérard F., Chabeaud A., Piot J. (2006). Antiproliferative activity of fish protein hydrolysates on human breast cancer cell lines. Process Biochem..

[265] Kannan A., hettiarachchy N.S., Marshall M., Raghavan S., Kristinsson H. (2011). Shrimp shell peptide hydrolysates inhibit human cancer cell proliferation. J. Scifood Agric..

[266] Wang Y.-K., He H.-L., Wang G.-F., Wu H., Zhou B.-C., Chen X.-L., Zhang Y.-Z. (2010). Oyster (*Crassostrea gigas*) hydrolysates produced on a plant scale have antitumor activity and immunostimulating effects in BALB/c mice. Mar. Drugs.

[267] Kim E.-K., Kim Y.-S., Hwang J.-W., Lee J.S., Moon S.-H., Jeon B.-T., Park P.-J. (2013). Purification and characterization of a novel anticancer peptide derived from *Ruditapes philippinarum*. Process Biochem..

[268] Wang X., Zhang X. (2013). Separation, antitumor activities, and encapsulation of polypeptide from *Chlorella pyrenoidosa*. Biotechnol. Prog..

[269] Hughes C.C., Fenical W. (2010). Antibacterials from the sea. Chem. A Eur. J..

[270] Zeng M., Liu Z., Zhao Y., Dong S., Kim S.K. (2013). Antimicrobial activities of marine protein and peptides. Marine Proteins Peptides: Biological Activities Applications.

[271] Smith V.J., Desbois A.P., Dyrynda E.A. (2010). Conventional and unconventional antimicrobials from fish, marine invertebrates and microalgae. Mar. Drugs.

[272] Cheung R.C.F., Ng T.B., Wong J.H. (2015). Marine peptides: Bioactivities and applications. Mar. Drugs.

[273] Beaulieu L., Bondu S., Doiron K., Rioux L.-E., Turgeon S.L. (2015). Characterization of antibacterial activity from protein hydrolysates of the macroalga Saccharina longicruris and identification of peptides implied in bioactivity. J. Funct. Foods.

[274] Campoverde C., Milne D.J., Estévez A., Duncan N., Secombes C.J., Andree K.B. (2017). Ontogeny and modulation after PAMPs stimulation of β-defensin, hepcidin, and piscidin antimicrobial peptides in meagre (*Argyrosomus regius*). Fish. Shellfish Immunol..

[275] Abdelhedi O., Nasri R., Mora L., Toldrá F., Nasri M., Jridi M. (2017). Collagenous proteins from black-barred halfbeak skin as a source of gelatin and bioactive peptides. Food Hydrocoll..

[276] Abuine R., Rathnayake A.U., Byun H.-G. (2019). Biological activity of peptides purified from fish skin hydrolysates. Fish. Aquat. Sci..

[277] Aissaoui N., Chobert J.-M., Haertlé T., Marzouki M.N., Abidi F. (2017). Purification and biochemical characterization of a neutral serine protease from *Trichoderma harzianum*: Use in antibacterial peptide production from a fish by-product hydrolysate. Appl. Biochem. Biotechnol..

[278] Guzmán F., Wong G., Román T., Cárdenas C., Alvárez C., Schmitt P., Albericio F., Rojas V. (2019). Identification of antimicrobial peptides from the microalgae *Tetraselmis suecica* (Kylin) Butcher and bactericidal activity improvement. Mar. Drugs.

[279] Kralovec J., Metera K., Kumar J., Watson L., Girouard G., Guan Y., Carr R., Barrow C., Ewart H. (2007). Immunostimulatory principles from *Chlorella pyrenoidosa*—Part 1: Isolation and biological assessment in vitro. Phytomedicine.

[280] Shaviklo A.R. (2015). Development of fish protein powder as an ingredient for food applications: A review. J. Food Sci. Technol..

[281] Park J.W. (2005). Surimi and Surimi Seafood.

[282] Kobayashi Y., Park J.W. (2018). Optimal blending of differently refined fish proteins based on their functional properties. J. Food Process. Preserv..

[283] Venugopal V. (2005). Seafood Processing: Adding Value through Quick Freezing, Retortable Packaging and Cook-Chilling.

[284] Shaviklo G.R., Olafsdottir A., Sveinsdottir K., Thorkelsson G., Rafipour F. (2011). Quality characteristics and consumer acceptance of a high fish protein puffed corn-fish snack. J. Food Sci. Technol..

[285] Adeleke R., Odedeji J. (2010). Acceptability studies on bread fortified with tilapia fish flour. Pak. J. Nutr..

[286] Ibrahim S. (2009). Evaluation of production and quality of salt-biscuits supplemented with fish protein concentrate. World J. Dairy Food Sci..

[287] Huda N., Abdullah A., Babji A.S. Substitution of Tapioca Flour with Surimi Powder in Traditional Crackers (Keropok Palembang). Proceedings of the 16th Scientific Conference Nutrition Society, Kuala Lumpu, Malaysia, 10–11 April 2001.

[288] Shaviklo A.R., Dehkordi A.K., Zangeneh P. (2014). Interactions and effects of the seasoning mixture containing fish protein powder/omega-3 fish oil on children’s liking and stability of extruded corn snacks using a mixture design approach. J. Food Process. Preserv..

[289] Supawong S., Park J.W., Thawornchinsombut S. (2018). Fat blocking roles of fish proteins in fried fish cake. LWT-Food Sci. Technol..

[290] Hashim P., Ridzwan M.M., Bakar J., Hashim M.D. (2015). Collagen in food and beverage industries. Int. Food Res. J..

[291] Subhan F., Ikram M., Shehzad A., Ghafoor A. (2015). Marine collagen: An emerging player in biomedical applications. J. Food Sci. Technol..

[292] Kim S.K. (2014). Marine cosmeceuticals. J. Cosmet. Dermatol..

[293] Pal G.K., Suresh P. (2016). Sustainable valorisation of seafood by-products: Recovery of collagen and development of collagen-based novel functional food ingredients. Innov. Food Sci. Emerg. Technol..

[294] Abdollahi M., Rezaei M., Jafarpour A., Undeland I. (2018). Sequential extraction of gel-forming proteins, collagen and collagen hydrolysate from gutted silver carp (*Hypophthalmichthys molitrix*), a biorefinery approach. Food Chem..

[295] Antoniewski M., Barringer S. (2010). Meat shelf-life and extension using collagen/gelatin coatings: A review. Crit. Rev. Food Sci. Nutr..

[296] Gómez-Guillén M., Giménez B., López-Caballero M.E., Montero M. (2011). Functional and bioactive properties of collagen and gelatin from alternative sources: A review. Food Hydrocoll..

[297] Karim A.A., Bhat R. (2008). Gelatin alternatives for the food industry: Recent developments, challenges and prospects. Trends Food Sci. Technol..

[298] Bilek S.E., Bayram S.K. (2015). Fruit juice drink production containing hydrolyzed collagen. J. Funct. Foods.

[299] Czajka A., Kania E.M., Genovese L., Corbo A., Merone G., Luci C., Sibilla S. (2018). Daily oral supplementation with collagen peptides combined with vitamins and other bioactive compounds improves skin elasticity and has a beneficial effect on joint and general wellbeing. Nutr. Res..

[300] Asserin J., Lati E., Shioya T., Prawitt J. (2015). The effect of oral collagen peptide supplementation on skin moisture and the dermal collagen network: Evidence from an ex vivo model and randomized, placebo-controlled clinical trials. J. Cosmet. Dermatol..

[301] Venkatesan J., Anil S., Kim S.-K., Shim M.S. (2017). Marine fish proteins and peptides for cosmeceuticals: A review. Mar. Drugs.

[302] Zhuang Y., Hou H., Zhao X., Zhang Z., Li B. (2009). Effects of collagen and collagen hydrolysate from jellyfish (*Rhopilema esculentum*) on mice skin photoaging induced by UV irradiation. J. Food Sci..

[303] Ehrlich H., Ehrlich H. (2015). Marine collagens. Biological Materials of Marine Origin.

[304] Lee C.H., Singla A., Lee Y. (2001). Biomedical applications of collagen. Int. J. Pharma..

[305] Yamada S., Yamamoto K., Ikeda T., Yanagiguchi K., Hayashi Y. (2014). Potency of fish collagen as a scaffold for regenerative medicine. Biomed. Res. Int..

[306] Olatunde O.O., Benjakul S. (2018). Natural preservatives for extending the shelf-life of seafood: A revisit. Compr. Rev. Food Sci. Food Saf..

[307] Etxabide A., Uranga J., Guerrero P., De la Caba K. (2017). Development of active gelatin films by means of valorisation of food processing waste: A review. Food Hydrocoll..

[308] Rawdkuen S., Suthiluk P., Kamhangwong D., Benjakul S. (2012). Mechanical, physico-chemical, and antimicrobial properties of gelatin-based film incorporated with catechin-lysozyme. Chem. Cent. J..

[309] Nuanmano S., Prodpran T., Benjakul S. (2015). Potential use of gelatin hydrolysate as plasticizer in fish myofibrillar protein film. Food Hydrocoll..

[310] Egerton S., Culloty S., Whooley J., Stanton C., Ross R.P. (2018). Characterization of protein hydrolysates from blue whiting (*Micromesistius poutassou*) and their application in beverage fortification. Food Chem..

[311] EFSA Panel on Dietetic Products, Nutrition and Allergies (2010). Scientific opinion on the safety of ‘sardine peptide product’. EFSA J..

[312] Lupo M.P., Cole A.L. (2007). Cosmeceutical peptides. Dermatol. Ther..

[313] Mourelle M.L., Gómez C.P., Legido J.L. (2017). The potential use of marine microalgae and cyanobacteria in cosmetics and thalassotherapy. Cosmetics.

[314] Hou H., Li B., Zhang Z., Xue C., Yu G., Wang J., Bao Y., Bu L., Sun J., Peng Z. (2012). Moisture absorption and retention properties, and activity in alleviating skin photodamage of collagen polypeptide from marine fish skin. Food Chem..

[315] Jimbo N., Kawada C., Nomura Y. (2016). Optimization of dose of collagen hydrolysate to prevent UVB-irradiated skin damage. Biosci. Biotechnol. Biochem..

[316] Sun L., Zhang Y., Zhuang Y. (2013). Antiphotoaging effect and purification of an antioxidant peptide from tilapia (*Oreochromis niloticus*) gelatin peptides. J. Funct. Foods.

[317] Just V. Bovine Collagen vs. Marine Collagen. https://www.justvitamins.co.uk/blog/bovine-collagen-vs-marine-collagen/#.

[318] Allard R., Malak N.A., Huc A. (2003). Collagen Product Containing Collagen of Marine Origin with a Low Odor and Preferably with Improved Mechanical Properties, and Its Use in the Form of Cosmetic or Pharmaceutical Compositions or Products. U.S. Patent.

[319] Bello A.E., Oesser S. (2006). Collagen hydrolysate for the treatment of osteoarthritis and other joint disorders: A review of the literature. Curr. Med. Res. Opin..

[320] Vellard M. (2003). The enzyme as drug: Application of enzymes as pharmaceuticals. Curr. Opin. Biotechnol..

[321] Kim S.-K., Dewapriya P., Kim S.-K. (2014). Enzymes from fish processing waste materials and their commercial applications. Seafood Processing by-Products. Trends and Applications.

[322] An H., Visessanguan W., Haard N.F., Simpson B.K. (2000). Recovery of enzymes from seafood-processing wastes. Seafood Enzymes: Utilisation and Influence on Postharvest Seafood Quality.

[323] Kandasamy N., Velmurugan P., Sundarvel A., Raghava R.J., Bangaru C., Palanisamy T. (2012). Eco-benign enzymatic dehairing of goatskins utilizing a protease from a *Pseudomonas fluorescens* species isolated from fish visceral waste. J. Clean. Prod..

[324] Klomklao S., Kishimura H., Yabe M., Benjakul S. (2007). Purification and characterization of two pepsins from the stomach of pectoral rattail (*Coryphaenoides pectoralis*). Com. Biochem. Physiol. Part B Biochem. Mol. Biol..

[325] Gudmundsdóttir Á., Pálsdóttir H.M. (2005). Atlantic cod trypsins: From basic research to practical applications. Mar. Biotechnol..

[326] Paul J. (1982). Isolation and characterization of a *Chlamydomonas* L-asparaginase. Biochem. J..

[327] Ebrahiminezhad A., Rasoul-Amini S., Ghoshoon M.B., Ghasemi Y. (2014). *Chlorella vulgaris*, a novel microalgal source for L-asparaginase production. Biocat. Agric. Biotechnol..

[328] Batool T., Makky E.A., Jalal M., Yusoff M.M. (2016). A comprehensive review on L-asparaginase and its applications. Appl. Biochem. Biotechnol..

[329] Bafana A., Dutt S., Kumar S., Ahuja P.S. (2011). Superoxide dismutase: An industrial perspective. Crit. Rev. Biotechnol..

[330] Undeland I., Kelleher S.D., Hultin H.O. (2002). Recovery of functional proteins from herring (*Clupea harengus*) light muscle by an acid or alkaline solubilization process. J. Agric. Food Chem..

[331] Hernández D., Molinuevo-Salces B., Riaño B., Larrán-García A.M., Tomás-Almenar C., García-González M.C. (2018). Recovery of protein concentrates from microalgal biomass grown in manure for fish feed and valorization of the by-products through anaerobic digestion. Front. Sustain. Food Syst..

[332] Ursu A.-V., Marcati A., Sayd T., Sante-Lhoutellier V., Djelveh G., Michaud P. (2014). Extraction, fractionation and functional properties of proteins from the microalgae *Chlorella vulgaris*. Bioresour. Technol..

[333] Shavandi A., Hu Z., Teh S., Zhao J., Carne A., Bekhit A., Bekhit A.E.-D.A. (2017). Antioxidant and functional properties of protein hydrolysates obtained from squid pen chitosan extraction effluent. Food Chem..

[334] Bourtoom T., Chinnan M., Jantawat P., Sanguandeekul R. (2009). Recovery and characterization of proteins precipitated from surimi wash-water. LWT-Food Sci. Technol..

[335] Kennedy J.F., Paterson M., Taylor D.W., Silva M.P., Gebelein C.G., Caraher C.E. (1994). Recovery of proteins from whey using chitosan as a coagulant. Biotechnology and Bioactive Polymer.

[336] Wibowo S., Velazquez G., Savant V., Torres J.A. (2007). Effect of chitosan type on protein and water recovery efficiency from surimi wash water treated with chitosan–alginate complexes. Bioresour. Technol..

[337] Holland C., Shahbaz M. (1985). The utilization of chitosan in mussel protein recovery. Ir. J. Food Sci. Technol..

[338] Trang S.T. Innovation of Fishery by-Products in Vietnam. Proceedings of the FFTC-KU Joint Seminar on Improved Utilization of Fishery by-Products as Potential Nutraceuticals and Functional Foods, Bangkok, Thailand, 25–29 October 2010.

[339] Chomnawang C., Yongsawatdigul J. (2013). Protein recovery of tilapia frame by-products by pH-shift method. J. Aquat. Food Prod. Technol..

[340] Ba F., Ursu A.V., Laroche C., Djelveh G. (2016). *Haematococcus pluvialis* soluble proteins: Extraction, characterization, concentration/fractionation and emulsifying properties. Bioresour. Technol..

[341] Garcia E.S., Van Leeuwen J., Safi C., Sijtsma L., Eppink M.H., Wijffels R.H., van den Berg C. (2018). Selective and energy efficient extraction of functional proteins from microalgae for food applications. Bioresour. Technol..

[342] Song K.-M., Jung S.K., Kim Y.H., Kim Y.E., Lee N.H. (2018). Development of industrial ultrasound system for mass production of collagen and biochemical characteristics of extracted collagen. Food Bioprod. Process..

[343] Mireles DeWitt C., Nabors R., Kleinholz C. (2007). Pilot plant scale production of protein from catfish treated by acid solubilization/isoelectric precipitation. J. Food Sci..

[344] Matak K.E., Tahergorabi R., Jaczynski J. (2015). A review: Protein isolates recovered by isoelectric solubilization/precipitation processing from muscle food by-products as a component of nutraceutical foods. Food Res. Int..

[345] Tahergorabi R., Beamer S.K., Matak K.E., Jaczynski J. (2012). Isoelectric solubilization/precipitation as a means to recover protein isolate from striped bass (*Morone saxatilis*) and its physicochemical properties in a nutraceutical seafood product. J. Agric. Food Chem..

[346] Gehring C., Gigliotti J., Moritz J., Tou J., Jaczynski J. (2011). Functional and nutritional characteristics of proteins and lipids recovered by isoelectric processing of fish by-products and low-value fish: A review. Food Chem..

[347] Benelhadj S., Gharsallaoui A., Degraeve P., Attia H., Ghorbel D. (2016). Effect of pH on the functional properties of *Arthrospira* (*Spirulina*) *platensis* protein isolate. Food Chem..

[348] Teuling E., Schrama J.W., Gruppen H., Wierenga P.A. (2019). Characterizing emulsion properties of microalgal and cyanobacterial protein isolates. Algal Res..

[349] Marmon S.K., Liljelind P., Undeland I. (2009). Removal of lipids, dioxins, and polychlorinated biphenyls during production of protein isolates from Baltic herring (*Clupea harengus*) using pH-shift processes. J. Agric. Food Chem..

[350] Petrova I., Tolstorebrov I., Eikevik T.M. (2018). Production of fish protein hydrolysates step by step: Technological aspects, equipment used, major energy costs and methods of their minimizing. Int. Aquat. Res..

[351] Pasupuleti V.K., Braun S., Pasupuleti V., Demain A. (2008). State of the art manufacturing of protein hydrolysates. Protein Hydrolysates in Biotechnology.

[352] Lahl W.J., Braun S.D. (1994). Enzymatic production of protein hydrolysates for food use. Food Technol..

[353] Howieson J., Choo K. (2017). New Opportunities for Seafood Processing Waste.

[354] Owen J., Mendoza I. (1985). Enzymatically hydrolyzed and bacterially fermented fishery product. J. Food Technol..

[355] Gildberg A., Espejo-Hermes J., Magno-Orejana F. (1984). Acceleration of autolysis during fish sauce fermentation by adding acid and reducing the salt content. J. Sci. Food Agric..

[356] Raa J., Gildberg A., Olley J.N. (1982). Fish silage: A review. Crit. Rev. Food Sci. Nutr..

[357] Van Wyk H.J., Heydenrych C.M. (1985). The production of naturally fermented fish silage using various lactobacilli and different carbohydrate sources. J. Sci. Food Agric..

[358] Arruda L.F.d., Borghesi R., Oetterer M. (2007). Use of fish waste as silage: A review. Braz. Arch. Biol. Technol..

[359] Hale M.B. (1972). Making Fish Protein Concentrate by Enzymatic Hydrolysis.

[360] Marti-Quijal F.J., Remize F., Meca G., Ferrer E., Ruiz M.-J., Barba F.J. (2020). Fermentation in fish and by-products processing: An overview of current research and future prospects. Curr. Opini. Food Sci..

[361] Onodenalore A.C., Shahidi F. (1996). Protein dispersions and hydrolysates from shark (*Isurus oxyrinchus*). J. Aquat. Food Prod. Technol..

[362] Välimaa A.-L., Mäkinen S., Mattila P., Marnila P., Pihlanto A., Mäki M., Hiidenhovi J. (2019). Fish and fish side streams are valuable sources of high-value components. Food Qual. Saf..

[363] Shahidi F., Han X.-Q., Synowiecki J. (1995). Production and characteristics of protein hydrolysates from capelin (*Mallotus villosus*). Food Chem..

[364] Kristinsson H.G., Rasco B.A. (2000). Kinetics of the hydrolysis of Atlantic salmon (*Salmo salar*) muscle proteins by alkaline proteases and a visceral serine protease mixture. Process Biochem..

[365] Beaulieu L., Thibodeau J., Bryl P., Carbonneau M.-É. (2009). Characterization of enzymatic hydrolyzed snow crab (*Chionoecetes opilio*) by-product fractions: A source of high-valued biomolecules. Bioresour. Technol..

[366] Beaulieu L., Thibodeau J., Bonnet C., Bryl P., Carbonneau M.-E. (2013). Evidence of anti-proliferative activities in blue mussel (*Mytilus edulis*) by-products. Mar. Drugs.

[367] Cheng S., Zhao J., Liu J., Wu J., Zhang Q. (2013). Technology for enzymolysis of jellyfish brain protein by bromelain. Agric. Sci. Technol..

[368] Yan M., Tao H., Qin S. (2016). Effect of enzyme type on the antioxidant activities and functional properties of enzymatic hydrolysates from sea cucumber (*Cucumaria frondosa*) viscera. J. Aquat. Food Prod. Technol..

[369] Ran X.G., Wang L.Y. (2014). Use of ultrasonic and pepsin treatment in tandem for collagen extraction from meat industry by-products. J. Sci. Food Agric..

[370] Marciniak A., Suwal S., Naderi N., Pouliot Y., Doyen A. (2018). Enhancing enzymatic hydrolysis of food proteins and production of bioactive peptides using high hydrostatic pressure technology. Trends Food Sci. Technol..

[371] Andler S.M., Goddard J.M. (2018). Transforming food waste: How immobilized enzymes can valorize waste streams into revenue streams. NPJ Sci. Food.

[372] De la Fuente B., Tornos A., Príncep A., Lorenzo J.M., Pateiro M., Berrada H., Barba F.J., Ruiz M.-J., Martí-Quijal F.J. (2020). Scaling-up processes: Patents and commercial applications. Advances in Food and Nutrition Research.

[373] He S., Franco C.M., Zhang W. (2015). Economic feasibility analysis of the industrial production of fish protein hydrolysates using conceptual process simulation software. J. Bioprocess. Biotech..

[374] Marnis, Syahrul, Fitri, Mardayulis (2016). Valuation of economic utilization of fish processing waste patin (*Pangasius hypopthalmus*) as an added value for fish processing industry players in the district Kampar, Riau. Int. J. Econ. Financ..

[375] Karayannakidis P.D., Chatziantoniou S.E., Zotos A. (2015). Co-extraction of gelatin and lipids from Yellowfin tuna (*Thunnus albacares*) skins: Physicochemical characterization, process simulation and economic analysis. J. Food Process. Pres..

[376] Nugraha A., Hardyastuti S., Mulyo J.H. (2017). Financial feasibility of Sijuk shrimp paste business in Sungai Padang village, Sijuk District, Belitung Regency. Agro Ekon..

[377] Asiedu A., Ben S., Resurreccion E., Kumar S. (2018). Techno-economic analysis of protein concentrate produced by flash hydrolysis of microalgae. Environ. Prog. Sustain..

[378] Torres-Acosta M.A., Ruiz-Ruiz F., Aguilar-Yáñez J.M., Benavides J., Rito-Palomares M. (2016). Economic analysis of pilot-scale production of B-phycoerythrin. Biotechnol. Prog..

[379] SAMPI SAMPI—Organically Certified Fish Hydrolysate. http://www.sampi.com.au/.

[380] Chen X.-L., Peng M., Li J., Tang B.-L., Shao X., Zhao F., Liu C., Zhang X.-Y., Li P.-Y., Shi M. (2017). Preparation and functional evaluation of collagen oligopeptide-rich hydrolysate from fish skin with the serine collagenolytic protease from *Pseudoalteromonas* sp. SM9913. Sci. Rep..

[381] Beal C.M., Hebner R.E., Webber M.E., Ruoff R.S., Seibert A.F. (2012). The energy return on investment for algal biocrude: Results for a research production facility. Bioenergy Res..

[382] Agyei D., Ongkudon C.M., Wei C.Y., Chan A.S., Danquah M.K. (2016). Bioprocess challenges to the isolation and purification of bioactive peptides. Food Bioprod. Process..

[383] Catherine N. Innovation in Processing and Product Development is Identifying New Opportunities to Increase the Value of Waste in the Seafood Sector. https://www.fishfiles.com.au/media/fish-magazine/FISH-Vol-24-3/New-value-from-seafood.

[384] Hua K., Cobcroft J.M., Cole A., Condon K., Jerry D.R., Mangott A., Praeger C., Vucko M.J., Zeng C., Zenger K. (2019). The future of aquatic protein: Implications for protein sources in aquaculture diets. One Earth.

[385] Chemat F., Khan M.K. (2011). Applications of ultrasound in food technology: Processing, preservation and extraction. Ultrasonic. Sonochem..

[386] Majid I., Nayik G.A., Nanda V. (2015). Ultrasonication and food technology: A review. Cogent Food Agric..

[387] Lebovka N., Vorobiev E., Chemat F. (2011). Enhancing Extraction Processes in the Food Industry.

[388] Tian J., Wang Y., Zhu Z., Zeng Q., Xin M. (2015). Recovery of tilapia (*Oreochromis niloticus*) protein isolate by high-intensity ultrasound-aided alkaline isoelectric solubilization/precipitation process. Food Bioprocess. Technol..

[389] Álvarez C., Lélu P., Lynch S.A., Tiwari B.K. (2018). Optimised protein recovery from mackerel whole fish by using sequential acid/alkaline isoelectric solubilization precipitation (ISP) extraction assisted by ultrasound. LWT-Food Sci. Technol..

[390] Álvarez C., Tiwari B.K. (2015). Ultrasound assisted extraction of proteins from fish processing by-products. Institute of Food Technologist.

[391] Kim H.K., Kim Y.H., Kim Y.J., Park H.J., Lee N.H. (2012). Effects of ultrasonic treatment on collagen extraction from skins of the sea bass *Lateolabrax japonicus*. Fish. Sci..

[392] Vernes L., Abert-Vian M., El Maâtaoui M., Tao Y., Bornard I., Chemat F. (2019). Application of ultrasound for green extraction of proteins from spirulina: Mechanism, optimization, modeling, and industrial prospects. Ultrason. Sonochem..

[393] Li D., Mu C., Cai S., Lin W. (2009). Ultrasonic irradiation in the enzymatic extraction of collagen. Ultrason. Sonochem..

[394] Waghmare A.G., Salve M.K., LeBlanc J.G., Arya S.S. (2016). Concentration and characterization of microalgae proteins from *Chlorella pyrenoidosa*. Bioresourc. Bioprocess..

[395] Hirata G.A.M., Bernardo A., Miranda E.A. (2010). Crystallization of porcine insulin with carbon dioxide as acidifying agent. Powder Technol..

[396] Nakamura K., Hoshino T., Ariyama H. (1991). Adsorption of carbon dioxide on proteins in the supercritical region. Agric. Biol. Chem..

[397] Chaitanya V., Senapati S. (2008). Self-assembled reverse micelles in supercritical CO_2_ entrap protein in native state. J. Am. Chem. Soc..

[398] Winters M.A., Knutson B.L., Debenedetti P.G., Sparks H.G., Przybycien T.M., Stevenson C.L., Prestrelski S.J. (1996). Precipitation of proteins in supercritical carbon dioxide. J. Pharm. Sci..

[399] Maheshwari P., Ooi E., Nikolov Z. (1995). Off-flavor removal from soy-protein isolate by using liquid and supercritical carbon dioxide. J. Am. Oil Chem. Soc..

[400] Bonnaillie L.M., Qi P., Wickham E., Tomasula P.M. (2014). Enrichment and purification of casein glycomacropeptide from whey protein isolate using supercritical carbon dioxide processing and membrane ultrafiltration. Foods.

[401] Khorshid N., Hossain M.M., Farid M. (2007). Precipitation of food protein using high pressure carbon dioxide. J. Food Eng..

[402] Yver A.L., Bonnaillie L.M., Yee W., McAloon A., Tomasula P.M. (2012). Fractionation of whey protein isolate with supercritical carbon dioxide—Process modeling and cost estimation. Int. J. Mol. Sci..

[403] Lima J., Seixas F., Coimbra J., Pimentel T., Barão C., Cardozo-Filho L. (2019). Continuous fractionation of whey protein isolates by using supercritical carbon dioxide. J. CO2 Utiliz..

[404] Park J.Y., Back S.S., Chun B.S. (2008). Protein properties of mackerel viscera extracted by supercritical carbon dioxide. Environ. Biol..

[405] Kang K.-Y., Ahn D.-H., Jung S.-M., Kim D.-H., Chun B.-S. (2005). Separation of protein and fatty acids from tuna viscera using supercritical carbon dioxide. Biotechno. Bioprocess. Eng..

[406] Zhou L., Zhang Y., Leng X., Liao X., Hu X. (2010). Acceleration of precipitation formation in peach juice induced by high-pressure carbon dioxide. J. Agric. Food Chem..

[407] Sarkari M., Darrat I., Knutson B.L. (2003). CO_2_ and fluorinated solvent-based technologies for protein microparticle precipitation from aqueous solutions. Biotechnol. Prog..

[408] Olano A., Calvo M.M., Troyano E., Amigo L. (1992). Changes in the fractions of carbohydrates and whey proteins during heat treatment of milk acidified with carbon dioxide. J. Dairy Res..

[409] Tomasula P., Boswell R., Dupre N. (1999). Buffer properties of milk treated with high pressure carbon dioxide. Milchwissenschaft.

[410] Tomasula P., Boswell R. (1999). Measurement of the solubility of carbon dioxide in milk at high pressures. J. Supercrit. Fluids.

[411] Moshashaée S., Bisrat M., Forbes R.T., Quinn é.Á., Nyqvist H., York P. (2003). Supercritical fluid processing of proteins: Lysozyme precipitation from aqueous solution. J. Pharm. Pharmacol..

[412] Hofland G., Berkhoff M., Witkamp G., Van der Wielen L. (2003). Dynamics of precipitation of casein with carbon dioxide. Int. Dairy J..

[413] Hofland G.W., de Rijke A., Thiering R., van der Wielen L.A., Witkamp G.-J. (2000). Isoelectric precipitation of soybean protein using carbon dioxide as a volatile acid. J. Chromatogr. B Biomed. Sci. Appl..

[414] Hofland G.W., van Es M., van der Wielen L.A., Witkamp G.-J. (1999). Isoelectric precipitation of casein using high-pressure CO_2_. Ind. Eng. Chem. Res..

[415] Perry R., DW G. (2007). Perry’s Chemical Engineers’ Handbook.

[416] Bonnaillie L.M., Tomasula P.M. (2012). Kinetics, aggregation behavior and optimization of the fractionation of whey protein isolate with hydrochloric acid. Food Bioprod. Process..

[417] Bonnaillie L.M., Tomasula P.M. (2012). Fractionation of whey protein isolate with supercritical carbon dioxide to produce enriched α-lactalbumin and β-lactoglobulin food ingredients. J. Agric. Food Chem..

[418] Zhong Q., Jin M. (2008). Enhanced functionalities of whey proteins treated with supercritical carbon dioxide. J. Dairy Sci..

